# Continuous glucose monitoring in pregnant women with type 1 diabetes (CONCEPTT): a multicentre international randomised controlled trial

**DOI:** 10.1016/S0140-6736(17)32400-5

**Published:** 2017-11-25

**Authors:** Denice S Feig, Lois E Donovan, Rosa Corcoy, Kellie E Murphy, Stephanie A Amiel, Katharine F Hunt, Elizabeth Asztalos, Jon F R Barrett, J Johanna Sanchez, Alberto de Leiva, Moshe Hod, Lois Jovanovic, Erin Keely, Ruth McManus, Eileen K Hutton, Claire L Meek, Zoe A Stewart, Tim Wysocki, Robert O'Brien, Katrina Ruedy, Craig Kollman, George Tomlinson, Helen R Murphy, CONCEPTT Collaborative Group, Jeannie Grisoni, Carolyn Byrne, Katy Davenport, Sandra Neoh, Claire Gougeon, Carolyn Oldford, Catherine Young, Louisa Green, Benedetta Rossi, Helen Rogers, Barbara Cleave, Michelle Strom, Juan María Adelantado, Ana Isabel Chico, Diana Tundidor, Janine Malcolm, Kathy Henry, Damian Morris, Gerry Rayman, Duncan Fowler, Susan Mitchell, Josephine Rosier, Rosemary Temple, Jeremy Turner, Gioia Canciani, Niranjala Hewapathirana, Leanne Piper, Anne Kudirka, Margaret Watson, Matteo Bonomo, Basilio Pintaudi, Federico Bertuzzi, Giuseppina Daniela, Elena Mion, Julia Lowe, Ilana Halperin, Anna Rogowsky, Sapida Adib, Robert Lindsay, David Carty, Isobel Crawford, Fiona Mackenzie, Therese McSorley, John Booth, Natalia McInnes, Ada Smith, Irene Stanton, Tracy Tazzeo, John Weisnagel, Peter Mansell, Nia Jones, Gayna Babington, Dawn Spick, Malcolm MacDougall, Sharon Chilton, Terri Cutts, Michelle Perkins, Eleanor Scott, Del Endersby, Anna Dover, Frances Dougherty, Susan Johnston, Simon Heller, Peter Novodorsky, Sue Hudson, Chloe Nisbet, Thomas Ransom, Jill Coolen, Darlene Baxendale, Richard Holt, Jane Forbes, Nicki Martin, Fiona Walbridge, Fidelma Dunne, Sharon Conway, Aoife Egan, Collette Kirwin, Michael Maresh, Gretta Kearney, Juliet Morris, Susan Quinn, Rudy Bilous, Rasha Mukhtar, Ariane Godbout, Sylvie Daigle, Alexandra Lubina, Margaret Jackson, Emma Paul, Julie Taylor, Robyn Houlden, Adriana Breen, Anita Banerjee, Anna Brackenridge, Annette Briley, Anna Reid, Claire Singh, Jill Newstead-Angel, Janet Baxter, Sam Philip, Martyna Chlost, Lynne Murray, Kristin Castorino, Donna Frase, Olivia Lou, Marlon Pragnell

**Affiliations:** a Department of Medicine, Sinai Health System, Toronto, ON, Canada; b Department of Obstetrics & Gynecology, Sinai Health System, Toronto, ON, Canada; c Lunenfeld-Tanenbaum Research Institute, Toronto, ON, Canada; d Department of Medicine, University of Toronto, Toronto, ON, Canada; e Department of Medicine, University of Calgary, Calgary, AB, Canada; f Department of Endocrinology and Nutrition, Hospital de la Santa Creu i Sant Pau CIBER-BBN, Barcelona, Spain; g Department of Women and Children's Health, St Thomas' Hospital, King's College London, London, UK; h Diabetes Research Group, Faculty of Life Sciences and Medicine, King's College London, London, UK; i Diabetes Service, Devision of Urgent Care, Planned Care and Allied Critical Services, King's College Hospital NHS Foundation Trust, London, UK; j Sunnybrook Research Institute, Toronto, ON, Canada; k Department of Obstetrics and Gynecology, Helen Schneider Hospital for Women, Rabin Medical Center, Petah, Tikvah, Israel; l Division of Endocrinology, University of Southern California, Los Angeles, CA, USA; m Department of Chemistry, University of California, Santa Barbara, CA, USA; n Department of Medicine, University of Ottawa, and The Ottawa Hospital, Ottawa, ON, Canada; o Department of Obstetrics & Gynecology, McMaster University Hamilton, ON, Canada; p Department of Medicine, St Joseph Health Care London, ON, Canada; q Department of Medicine, University of Western ON, London, ON, Canada; r Wolfson Diabetes and Endocrine Centre, Cambridge University Hospitals NHS Foundation Trust, Cambridge, UK; s Nemours Children's Health System, Jacksonville, FL, USA; t Jaeb Center For Health Research, Tampa, FL, USA; u Department of Medicine, University Health Network, Toronto, ON, Canada; v Department of Medicine, University of East Anglia, Norwich, UK

## Abstract

**Background:**

Pregnant women with type 1 diabetes are a high-risk population who are recommended to strive for optimal glucose control, but neonatal outcomes attributed to maternal hyperglycaemia remain suboptimal. Our aim was to examine the effectiveness of continuous glucose monitoring (CGM) on maternal glucose control and obstetric and neonatal health outcomes.

**Methods:**

In this multicentre, open-label, randomised controlled trial, we recruited women aged 18–40 years with type 1 diabetes for a minimum of 12 months who were receiving intensive insulin therapy. Participants were pregnant (≤13 weeks and 6 days' gestation) or planning pregnancy from 31 hospitals in Canada, England, Scotland, Spain, Italy, Ireland, and the USA. We ran two trials in parallel for pregnant participants and for participants planning pregnancy. In both trials, participants were randomly assigned to either CGM in addition to capillary glucose monitoring or capillary glucose monitoring alone. Randomisation was stratified by insulin delivery (pump or injections) and baseline glycated haemoglobin (HbA_1c_). The primary outcome was change in HbA_1c_ from randomisation to 34 weeks' gestation in pregnant women and to 24 weeks or conception in women planning pregnancy, and was assessed in all randomised participants with baseline assessments. Secondary outcomes included obstetric and neonatal health outcomes, assessed with all available data without imputation. This trial is registered with ClinicalTrials.gov, number NCT01788527.

**Findings:**

Between March 25, 2013, and March 22, 2016, we randomly assigned 325 women (215 pregnant, 110 planning pregnancy) to capillary glucose monitoring with CGM (108 pregnant and 53 planning pregnancy) or without (107 pregnant and 57 planning pregnancy). We found a small difference in HbA_1c_ in pregnant women using CGM (mean difference −0·19%; 95% CI −0·34 to −0·03; p=0·0207). Pregnant CGM users spent more time in target (68% *vs* 61%; p=0·0034) and less time hyperglycaemic (27% *vs* 32%; p=0·0279) than did pregnant control participants, with comparable severe hypoglycaemia episodes (18 CGM and 21 control) and time spent hypoglycaemic (3% *vs* 4%; p=0·10). Neonatal health outcomes were significantly improved, with lower incidence of large for gestational age (odds ratio 0·51, 95% CI 0·28 to 0·90; p=0·0210), fewer neonatal intensive care admissions lasting more than 24 h (0·48; 0·26 to 0·86; p=0·0157), fewer incidences of neonatal hypoglycaemia (0·45; 0·22 to 0·89; p=0·0250), and 1-day shorter length of hospital stay (p=0·0091). We found no apparent benefit of CGM in women planning pregnancy. Adverse events occurred in 51 (48%) of CGM participants and 43 (40%) of control participants in the pregnancy trial, and in 12 (27%) of CGM participants and 21 (37%) of control participants in the planning pregnancy trial. Serious adverse events occurred in 13 (6%) participants in the pregnancy trial (eight [7%] CGM, five [5%] control) and in three (3%) participants in the planning pregnancy trial (two [4%] CGM and one [2%] control). The most common adverse events were skin reactions occurring in 49 (48%) of 103 CGM participants and eight (8%) of 104 control participants during pregnancy and in 23 (44%) of 52 CGM participants and five (9%) of 57 control participants in the planning pregnancy trial. The most common serious adverse events were gastrointestinal (nausea and vomiting in four participants during pregnancy and three participants planning pregnancy).

**Interpretation:**

Use of CGM during pregnancy in patients with type 1 diabetes is associated with improved neonatal outcomes, which are likely to be attributed to reduced exposure to maternal hyperglycaemia. CGM should be offered to all pregnant women with type 1 diabetes using intensive insulin therapy. This study is the first to indicate potential for improvements in non-glycaemic health outcomes from CGM use.

**Funding:**

Juvenile Diabetes Research Foundation, Canadian Clinical Trials Network, and National Institute for Health Research.

## Introduction

Type 1 diabetes increases the risk of adverse pregnancy outcomes, including higher rates of pre-eclampsia and caesarean section in mothers, and of congenital anomaly, preterm delivery, perinatal mortality, large for gestational age, and neonatal intensive care admission in infants.^1^, ^2^, ^3^, ^4^ The evidence that optimal glycaemic control early during the first trimester is associated with improved outcomes, reduced congenital anomalies and perinatal mortality is well established.^5^, ^6^ Likewise, reduced exposure to maternal hyperglycaemia during the second and third trimesters is associated with reduced pre-eclampsia, preterm delivery, large for gestational age, and neonatal intensive care admissions.^7^, ^8^, ^9^

**Research in context**

**Evidence before this study**

We searched PubMed for articles published before June 30, 2017, without restriction on language or start date. We included the search terms (“Diabetes Mellitus” OR “Diabetes”), AND “pregnancy”, OR “pregnancy in diabetics”, AND “continuous glucose monitoring”, AND (“trial or randomised controlled trial”). We identified three randomised trials that used continuous glucose monitoring in women with pre-existing diabetes during pregnancy, which found conflicting results. Two of the three studies included women with both type 1 and type 2 diabetes. One study found a reduction in glycated haemoglobin (HbA_1c_) and macrosomia; however, it used an intermittent, masked continuous glucose monitoring (CGM) device so that patients did not see the glucose values in real time. Another study used intermittent real-time CGM and found no difference in glycaemic control or perinatal outcomes. However, only five women in this study used real-time CGM continuously. Together, these two studies included 225 women of whom 165 had type 1 diabetes. A third small pilot study compared continuous and intermittent CGM in 25 women with type 1 diabetes, and found no difference in glycaemic control or neonatal outcomes; however, the study was not powered for these outcomes. A 2017 Cochrane review of self-blood glucose monitoring among pregnant women with pre-existing diabetes concluded that the evidence is weak regarding the efficacy of continuous glucose monitoring and that additional evidence from well designed, large randomised trials is needed to inform choices of glucose monitoring.

**Added value of this study**

In our multicentre, international, randomised controlled trial in 325 women with type 1 diabetes, we randomly assigned women during early pregnancy or planning pregnancy to receive either real-time CGM or standard capillary glucose monitoring. We found a small but significant reduction in HbA_1c_ levels at 34 weeks' gestation, accompanied by an increased time in target, reduced hyperglycaemia, and less glycaemic variability. This was accompanied by reductions in neonatal outcomes in the proportion of infants large for gestational age, neonatal hypoglycaemia, admission to neonatal intensive care for more than 24 h, and a 1-day shorter hospital stay among infants of mothers randomly assigned to CGM during the first trimester. The results were generalisable across 31 international study sites and comparable for women using insulin pumps or multiple daily injections, regardless of baseline glucose control. By contrast, we found no significant benefit from CGM use in women planning pregnancy.

**Implications of all the available evidence**

Our study indicates that the use of CGM during pregnancy in women with type 1 diabetes is associated with improved neonatal health outcomes attributed to reduced exposure to maternal hyperglycaemia. The numbers of pregnant women needed to treat with CGM to prevent one newborn complication are six for both neonatal intensive care admission and large for gestational age, and eight for neonatal hypoglycaemia. National and international clinical guideline recommendations in type 1 diabetes in pregnancy should be revised to recommend offering CGM to pregnant women with type 1 diabetes using intensive insulin therapy in the first trimester. To our knowledge, this study is the first to indicate potential for improvements in non-glycaemic health outcomes from CGM use.

Thus, clinical care guidelines recommend that women with type 1 diabetes strive for optimal glycaemic control before and during pregnancy.^10^ However, this optimal glycaemic control is difficult to accomplish given the complexity of insulin dose adjustment, gestational changes in insulin sensitivity and marked day-to-day variability in insulin absorption during late pregnancy.^11^, ^12^, ^13^ Nationwide UK data confirm that only 15% of pregnant women achieve target glycated haemoglobin (HbA_1c_) levels during early pregnancy and that despite intensive support (2-weekly antenatal clinics and frequent between-clinic contacts), only 40% of women with type 1 diabetes achieve target HbA_1c_ levels after 24 weeks' gestation.^9^ Thus one in two newborn infants experience complications associated with exposure to maternal hyperglycaemia, with no improvement in neonatal morbidity in the past three to four decades.^9^ Furthermore, as women strive for optimal glucose control, the rates of severe hypoglycaemia, particularly during early pregnancy, are five times higher than in non-pregnant women.^14^

Continuous glucose monitoring (CGM) provides detailed data on the direction and rate of change of glucose levels.^15^. Real-time systems display contemporaneous glucose readings, enabling users to respond to changes as they occur. Although randomised trials in motivated, non-pregnant women suggest that regular use of CGM, defined as at least 6 days per week, improves glycaemic control,^16^, ^17^ results in pregnant women have been conflicting. Previous studies provided data only for intermittent retrospective use or for early generation systems that were too inaccurate or uncomfortable for regular use.^18^, ^19^ The role of real-time CGM used throughout pregnancy has not been established and no studies have included women planning pregnancy. Beyond glucose control, no studies, either in pregnant or non-pregnant populations, have examined the effect on diabetes complications or other health outcomes. Our aim was to evaluate the effectiveness of CGM used before and from early pregnancy on maternal glucose control obstetric outcomes and neonatal health outcomes.

## Methods

### Study design and participants

This open-label, multicentre, multinational, randomised, controlled study included two parallel trials: a pregnancy trial and a planning pregnancy trial. Participants were recruited from 31 hospitals in Canada, England, Scotland, Spain, Italy, Ireland, and the USA.

The clinical study protocol was approved by the Health Research Authority, East of England Research Ethics Committee (12/EE/0310) for all UK sites and at each individual centre for all other sites. All participants provided written informed consent. Full details of the clinical study protocol have been previously published.^20^

We recruited women aged 18–40 years with type 1 diabetes for a minimum of 12 months, receiving intensive insulin therapy via multiple daily injections or an insulin pump, who were pregnant or planning pregnancy. Pregnant women were eligible if they had a live singleton fetus confirmed by ultrasound, were at 13 weeks and 6 days' gestation or less, and had HbA_1c_ between 6·5–10·0% (48–86 mmol/mol). Women planning for pregnancy were eligible if they had an HbA_1c_ level between 7·0–10·0% (53–86 mmol/mol).

Regular CGM users and women with severe nephropathy or medical conditions such as psychiatric illness requiring hospitalisation that could prevent them from completing the trial were excluded. Women using automatic insulin delivery options, such as low glucose suspend pumps, were not excluded.

### Randomisation and masking

After enrolment, participants had to complete a run-in phase with a masked CGM device (iPro2 Professional CGM, Medtronic, Northridge, CA, USA) before they were eligible for randomisation. In the run-in period, glucose values were recorded but were not visible to the user or clinical team. Eligibility required that participants wear the sensor for 6 days, provide at least 96 h of glucose values including a minimum of 24 h overnight, and obtain at least four capillary glucose tests daily. Participants meeting these criteria were randomised to receive either CGM in addition to capillary glucose monitoring (intervention) or capillary glucose monitoring alone (control). Treatments were allocated in a 1:1 ratio via a web-based system that used a computer-generated randomisation list with permuted block sizes and stratification by method of insulin delivery (pump or multiple injections), and baseline HbA_1c_ (<7·5% *vs* ≥7·5% or 58 mmol/mol for the pregnancy trial; <8·0% *vs* ≥8·0% or 64 mmol/mol for the planning pregnancy trial). The randomisation schedule was created by a programming manager, encrypted, and maintained in a secure database to which the trial coordinating team and investigators had no access.

### Procedures

Participants in the CGM group were provided with a CGM system (Guardian REAL-Time or MiniMed Minilink system, both Medtronic, Northridge, CA). They were trained to use the study devices and were instructed to use them daily by their local diabetes or antenatal clinical teams. CGM users were advised to verify the accuracy of CGM measurements using their capillary glucose meter before insulin dose adjustment, as per the regulatory labelling instructions. Participants in the control group continued their usual method of capillary glucose monitoring. Participants in both groups were advised to test capillary glucose levels at least seven times daily (before and 1–2 h after meals and before bed) and given written instructions for how to use capillary or CGM measures for insulin dose adjustment, customised for method of insulin delivery. The detailed treatment algorithms have been published previously.^20^ Both groups had the same glucose target range of 3·5–7·8 mmol/L and same target HbA_1c_ levels of no higher than 6·5% (48 mmol/mol) during pregnancy and no higher than 7·0% (53 mmol/mol) if planning pregnancy.

In the pregnancy trial, study visits were scheduled at randomisation (≤13 weeks and 6 days' gestation), and at 8, 12, 16, 20, 24, 28, 32, 34, and 36 weeks' gestation. In the planning pregnancy trial, study visits were scheduled at 4, 8, 12, 16, 20, and 24 weeks after randomisation. Women who conceived during the trial continued in their same randomised group and followed the pregnancy study visit schedule. Participants' weights, blood pressure, insulin dose, adverse events, and episodes of severe hypoglycaemia (defined as an event requiring third party assistance) were recorded at each visit. Additional clinic visits or telephone or email contacts would occur if they were part of the centres' usual care protocols or if indicated by clinical need. All scheduled and unscheduled (telephone or email) contacts were documented in the electronic clinical report forms.

HbA_1c_ measures were taken at randomisation, 24 weeks' gestation, and 34 weeks' gestation in the pregnancy trial, and at randomisation, 12 weeks, and 24 weeks in the planning pregnancy trial; if a participant conceived before 24 weeks, their final HbA_1c_ measure was taken following confirmation of a positive pregnancy test. All HbA_1c_ measurements were done using the turbidimetric inhibition immunoassay for haemolysed whole blood on the Cobas Integra 700 platform (Roche, Basel, Switzerland) at a central laboratory (DynaCare, Brampton, ON, Canada). The samples were shipped at the end of the planning pregnancy trial or after delivery of the neonate and collection of the cord blood and were unavailable to participants and health-care teams during the trial. After the visits at 24 and 34 weeks' gestation in the pregnancy trial, and 12 and 24 weeks or following confirmation of a positive pregnancy test in the planning pregnancy trial, CGM measures were obtained using real-time sensors in the CGM group and a masked sensor in the control group. These were repeated, as required, if fewer than 96 h of glucose values were obtained.

### Outcomes

The primary outcome was difference in change in HbA_1c_ from randomisation to 34 weeks' gestation in the pregnancy trial and to 24 weeks or conception in the planning pregnancy trial.

Prespecified secondary glycaemic outcomes for all groups were percentage of time spent in, above, and below the recommended glucose control target range (3·5–7·8 mmol/L); area under the curve for glucose levels; episodes of hypoglycaemia; and glucose variability measures derived from CGM measures. For pregnant women, prespecified health outcomes were gestational weight gain, gestational hypertension, pre-eclampsia, mode of delivery, length of hospital stay, insulin dose, and questionnaires relating to fear of hypoglycaemia, coping with diabetes, quality of life, and satisfaction with monitoring device. Prespecified neonatal health outcomes included preterm delivery, neonatal hypoglycaemia requiring intravenous dextrose, neonatal intensive care unit admission requiring a duration of at least 24 h, cord blood gas pH, total length of hospital stay, birthweight, and macrosomia (birthweight ≥4 kg). Because of differences in gestational age at delivery, we prespecified use of customised birthweight percentiles (gestation-related optimal weight) that adjust for infant sex and gestational age as well as maternal height, weight, parity, and ethnicity to calculate the birthweight percentile and proportion of infants large or small for gestational age (birthweight percentile >90th or <tenth).^21^

We included the following outcomes both as individual and as a composite neonatal measure: pregnancy loss (miscarriage, stillbirth, or neonatal death), birth injury, shoulder dystocia, neonatal hypoglycaemia, hyperbilirubinaemia, respiratory distress syndrome, or neonatal intensive care admission. To capture clinically important adverse outcomes, neonatal hypoglycaemia was defined as requiring treatment with intravenous dextrose and neonatal intensive care unit admission as requiring a duration of at least 24 h. We measured fetal hyperinsulinaemia and infant adiposity with cord blood C-peptide, anthropometric measurements, sum of skin-folds, and neonatal fat mass.^22^

At baseline and trial completion, participants completed the following questionnaires: Blood Glucose Monitoring System Rating Questionnaire (BGMSRQ),^23^ Problem Areas in Diabetes (PAID),^24^ Short-Form-12,^25^ and Hypoglycaemia Fear Survey (HFS II).^26^ At follow-up, CGM participants completed an additional questionnaire on their satisfaction with CGM and its effects on their lifestyle and behaviour.^27^ Complications, including episodes of diabetic ketoacidosis, severe hypoglycaemia, hospital admissions for diabetes, and those common to pregnancy with type 1 diabetes including hospital admissions for obstetric indications and adverse pregnancy outcomes (congenital anomaly, stillbirth, neonatal death), were identified and recorded as outcomes.

Adverse events were captured throughout the study. Reportable adverse events included all serious adverse events other than prespecified protocol exceptions. Maternal death was considered a serious adverse event regardless of whether or not it was caused by severe hypoglycaemia.

### Statistical analysis

To achieve 90% power at a two-sided 5% significance level, we planned a sample size of 214 pregnant participants and 110 participants planning pregnancy to detect a between-group difference in HbA_1c_ of 0·5%. We assumed a correlation of 0·4 between baseline and follow-up HbA_1c_, with an SD of 1·1 and up to 20% loss to follow-up during pregnancy; and an SD of 0·8 and 15% loss to follow-up before pregnancy.

The primary analysis used ANCOVA to compare treatment arms with adjustment for baseline HbA_1c_ and insulin therapy (pump or multiple daily injections). We included all randomised women who had a baseline assessment, with multiple imputation of missing HbA_1c_ values using the mice package in R.^28^ For binary outcomes, we compared the proportions between groups using Fisher's exact test and, where applicable, using regression models that allowed for varying lengths of follow-up time and the possibility of overdispersion. We used repeated measures ANOVA to determine the significance of differences in total scores for patient-reported outcome measures as estimated by main effects for groups (CGM *vs* control) and group × time interaction effects. To avoid potential baseline imbalances, we did not include the obstetric and neonatal outcomes of women in the planning pregnancy trial who conceived with those of the pregnancy trial. Analyses of secondary outcomes used all available data without imputation. We used a two-sided significance level of 5% for primary and secondary outcomes without adjustment for multiple comparisons.

We used logistic regression analyses to estimate the odds of occurrence of an adverse event with 95% CIs by intervention versus control group. We used Poisson regression to calculate the rate of occurrence 95% CIs over the study period (randomisation until delivery). p values are from these models, with baseline HbA_1c_ group and method of insulin delivery as covariates.

The trial was overseen by a trial steering committee and an independent data safety monitoring board. An interim safety review (maternal severe hypoglycaemia and neonatal outcomes) was done when 50% of neonates were delivered. This trial is registered with ClinicalTrials.gov. number NCT01788527.

### Role of the funding source

The funders had no role in the trial design, data collection, data analysis, or data interpretation. Medtronic (Northridge, CA, USA) provided discounted-price insulin pumps and CGM devices. They also had no role in trial design, collection, handling, analysis, or interpretation of data, or the decision to publish. The National Institute for Health Research and Juvenile Diabetes Research Foundation reviewed the manuscript prior to submission but did not play a part in manuscript preparation or revision. DSF and HRM oversaw the conduct of the trial, had full access to all the data, and take full responsibility for the decision to submit for publication.

## Results

**Figure 1:**
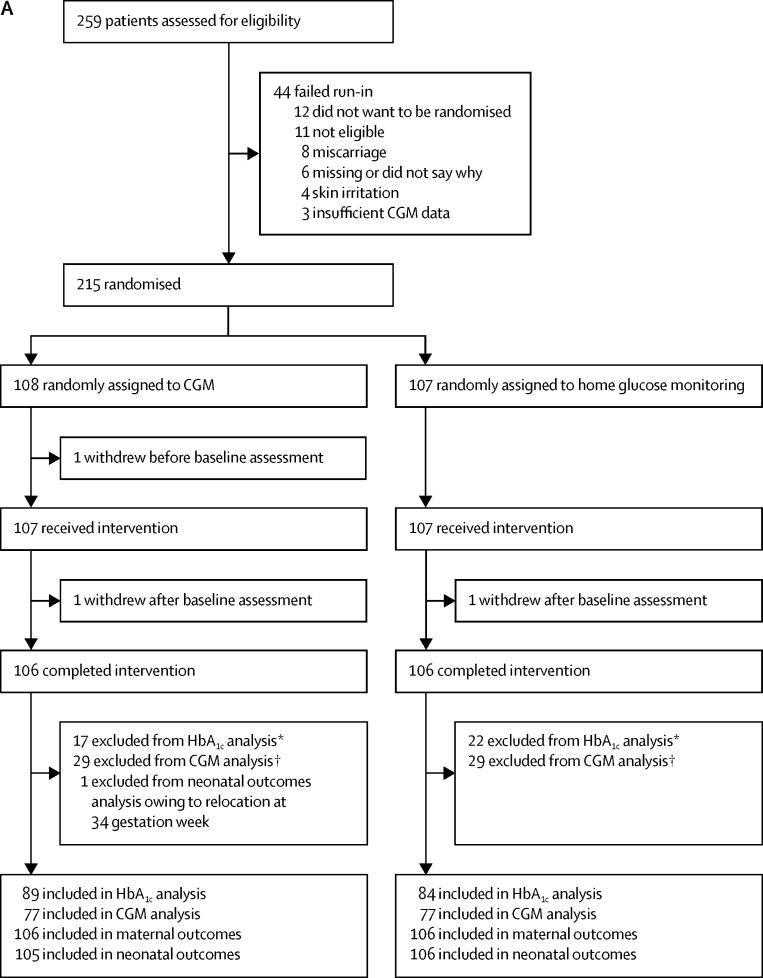

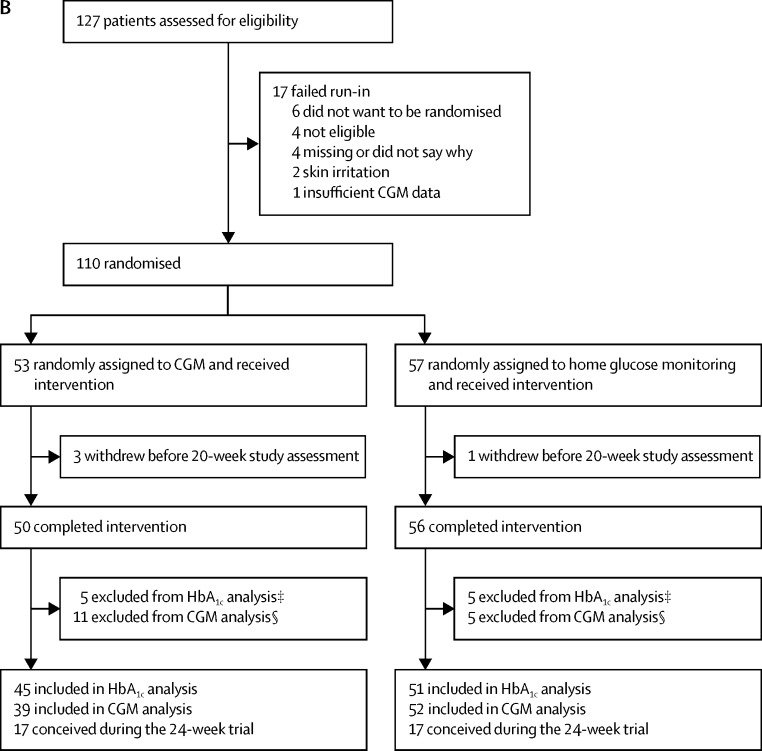
Trial profile for (A) participants in the pregnancy trial and (B) participants in the planning pregnancy trial CGM=continuous glucose monitoring. HbA_1c_= glycated haemoglobin. *Central laboratory HbA_1c_ data were not available at randomisation for nine CGM group participants (six lost or not collected, two withdrawals, and one pregnancy loss) and 11 control group participants (nine lost or not collected, one withdrawal, and one pregnancy loss) and not available for an additional ten participants in the CGM group and 12 participants in the control group at 34 weeks. †CGM data unavailable. CGM data were available for 77 CGM participants using real-time sensor and 77 control group participants using an iPro2 masked sensor. ‡Central laboratory HbA_1c_ measurements were not available for eight participants in the CGM group (five lost or not collected and three withdrawals) and six control group participants (five lost or not collected and one withdrawal). §CGM data not available. CGM data were available for 39 CGM participants using real-time sensor and 52 control group participants using an iPro2 masked sensor.

Between March 25, 2013, and March 22, 2016, 386 participants were assessed for eligibility and 325 participants were randomised, with 215 pregnant and 110 planning pregnancy. In the pregnancy trial, 108 women were assigned to the CGM intervention and 107 women were assigned to the control group. One CGM participant withdrew before the baseline assessments, leaving 107 in each group (figure 1A). In the planning pregnancy trial, 53 women were assigned to the intervention and 57 to the control group (figure 1B). Most participants self-identified as of European or Mediterranean origin, were college educated, non-smokers, and had a long duration of type 1 diabetes (table 1). Approximately half were overweight or obese. Half the women in the pregnancy trial took folic acid preconception and slightly more than half used multiple daily insulin injections. In the planning pregnancy trial, a greater proportion of women used insulin pump therapy than in the pregnancy trial. The mean HbA_1c_ levels at randomisation were lower in the pregnant women (table 1). Any minor imbalances in baseline characteristics between CGM and control group participants were within the expected bounds for random allocation.

**Table 1:** Baseline characteristics of participants according to pregnancy status

		**Pregnant**		**Planning pregnancy**	
		CGM (n=108)*	Control (n=107)	CGM (n=53)	Control (n=57)
Age (years)		31·4 (4·5)	31·5 (4·9)	33·5 (3·5)	32·4 (3·6)
European origin		94 (87%)	90 (84%)	44 (83%)	51 (89%)
Gestational age (weeks)		10·5 (2·2)	11·0 (2·0)	··	··
Primiparous		44/107 (41%)	40 (37%)	··	··
Body-mass index (kg/m^2^)†		26·1 (5·1)	25·3 (3·8)	26·4 (4·2)	26·6 (4·9)
	Normal (<25 kg/m^2^)	53/107 (50%)	56 (52%)	23 (43%)	23 (40%)
	Overweight (25–30 kg/m^2^)	33/107 (31%)	38 (36%)	23 (43%)	21 (37%)
	Obese (≥30 kg/m^2^)	21/107 (20%)	13 (12%)	7 (13%)	12 (21%)
Duration of diabetes (years)		17·0 (6·0–28·0)	16·0 (6·6–26·4)	18·0 (6·2–30·0)	19·0 (9·0–28·0)
HbA_1c_ at enrolment‡					
	Percentage	7·43 (0·70)	7·37 (0·77)	7·91 (0·69)	7·85 (0·67)
	mmol/mol	58 (7·3)	57 (8·4)	63 (7·5)	62 (7·3)
HbA_1c_ at randomisation§					
	Percentage	6·83 (0·67)	6·95 (0·66)	7·57% (0·77)	7·57 (0·58)
	mmol/mol	51 (7·3)	52 (7·2)	59 (8·4)	59 (6·3)
Smoking		13/107 (12%)	23 (21%)	3 (6%)	6 (11%)
Post-secondary education		88 (81%)	77 (72%)	46 (87%)	47 (82%)
Pre-conception folic acid		54/107 (50%)	55 (51%)	29 (55%)	27 (47%)
Pre-conception multivitamin		35/107 (33%)	26 (24%)	18 (34%)	19 (33%)
Insulin pump		50 (46%)	48 (45%)	39 (74%)	42 (74%)
Automated insulin delivery option¶		19/103 (18%)	6/104 (6%)	6/52 (11%)	1 (2%)
Insulin injections		58 (54%)	59 (55%)	14 (26%)	15 (26%)
Total insulin dose (U/kg per day)		0·69 (0·25)	0·76 (0·31)	0·61 (0·19)	0·61 (0·16)
Diabetes complications||		27 (25%)	30 (28%)	18 (34%)	23 (40%)
	Retinopathy	22	29	16	19
	Nephropathy	6	2	3	3
	Neuropathy	3	4	2	4
Hypertension		4 (4%)	10 (9%)	11 (21%)	7 (12%)
Systolic blood pressure (mm Hg)		116·0 (14·0)	116·0 (13·6)	118·8 (13·8)	116·2 (12·0)
Diastolic blood pressure (mm Hg)		69·1 (8·8)	70·0 (8·6)	73·7 (8·4)	71·7 (8·1)
Severe hypoglycaemia in past year**		13/107 (12%)	7 (7%)	3 (6%)	7 (12%)
Severe hypoglycaemia during early pregnancy (pre-randomisation)		7/107 (7%)	4 (4%)	··	··
Hypoglycaemia awareness symptoms					
	Always aware	74/107 (69%)	76 (71%)	42 (79)	48 (84%)
	Sometimes	30/107 (28%)	28 (26%)	10 (19%)	8 (14%)
	Never aware	3/107 (3%)	3 (3%)	1 (2%)	1 (2%)

Data are mean (SD), n (%), or n/N (%) where data are missing, except for duration of diabetes, which is median (tenth to 90th percentile). Data were collected at enrolment or randomisation (2 weeks after enrolment). CGM=continuous glucose monitoring. HbA_1c_= glycated haemoglobin.

* One CGM participant withdrew immediately after randomisation and before baseline assessments, leaving 107 in both planning-pregnancy groups.

† One underweight control participant (body-mass index <18·5 kg/m^2^) was planning pregnancy.

‡ Locally assessed.

§ Centrally assessed. Randomisation HbA_1c_ levels were unavailable for nine CGM participants (six lost or not collected, two withdrawals, and one pregnancy loss) and 11 control participants (nine lost or not collected, one withdrawal, and one pregnancy loss) in early pregnancy and for seven CGM participants (four lost or not collected, two withdrawals, and one pregnant) and five control participants (five lost or not collected) planning pregnancy. All participants had HbA_1c_ levels (local lab) at enrolment.

¶ 25 pregnant participants and seven participants planning pregnancy used pumps with low glucose suspend features. Data regarding the use or frequency of insulin suspension is not available.

|| Diabetes complications are not mutually exclusive.

** Severe hypoglycaemia was defined as an episode requiring third-party assistance.

The proportion of completed scheduled study visits was high. Pregnant participants using CGM completed slightly more scheduled visits than did the control group (mean 7·2 [SD 1·1] *vs* 6·8 [1·4]; p=0·0171), whereas we found no between-group differences in participants planning pregnancy. Pregnant participants using CGM also had more unscheduled contacts (1530 contacts by CGM participants *vs* 1026 contacts by controls; appendix p 3) outside of their study and usual care visits. These unscheduled contacts were attributed both to sensor issues (mean 2·1 [SD 2·8] per participant in the CGM group *vs* 0·1 [0·3] per participant in the control group; p<0·0001) and to sensor-related diabetes management issues (2·6 [5·3] *vs* 0·2 [SD 0·7]; p<0·0001). We found no difference in contacts for general diabetes management or contacts for other reasons. We noted similar findings among CGM users in the planning pregnancy trial (appendix p 3).

The frequency of CGM use was comparable in pregnant participants (median 6·1 days per week [IQR 4·0–6·8]) and participants planning pregnancy (6·2 days per week [IQR 5·2–6·9]). Sensor compliance was generally high with 70% of pregnant participants and 77% of participants planning pregnancy using CGM for more than 75% of the time. Sensor use was highest in later gestation (median 6·5 days [IQR 3·9–7·0] at 25–34 weeks) in pregnant women and earlier (median 6·7 days [IQR 5·3–6·9] at 1–12 weeks after randomisation) in women planning pregnancy (appendix p 4).

**Figure 2:**
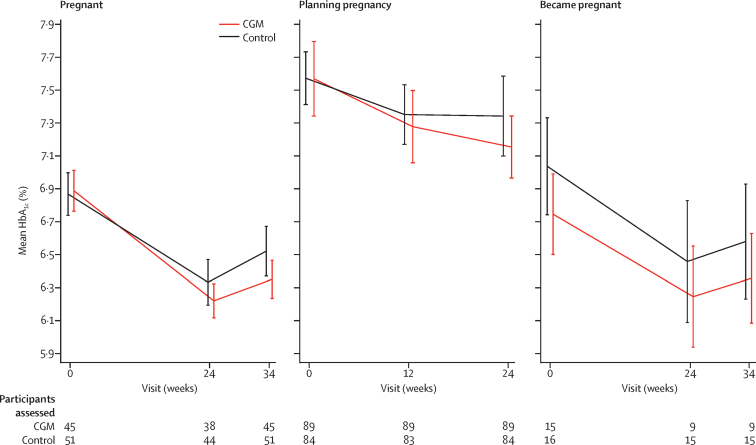
Primary glycaemic outcome showing participants' HbA_1c_ levels according to pregnancy status Mean HbA_1c_ (95% CI) is shown at each assessment time for participants who had data at baseline and the time of the outcome assessment (24 and 34 weeks' gestation in the pregnancy trial and at 12 and 24 weeks from randomisation or at time of confirmed pregnancy in the pregnancy planning trial). Data are also shown for participants in the planning pregnancy trial who conceived before 24 weeks and stayed in the trial during pregnancy. CGM=continuous glucose monitoring. HbA_1c_=glycated haemoglobin.

In the primary analysis of the pregnancy trial, we found a small but significant between-group difference in the change in HbA_1c_ from baseline to 34 weeks' gestation, favouring CGM (mean difference −0·19%, 95% CI −0·34 to −0·03; p=0·0207; figure 2, table 2. In the planning pregnancy trial, the between-group difference was of a similar size but with a wider confidence interval and not significant (−0·17%, 95% CI −0·43 to 0·09; p=0·20; figure 2). Outcomes of the 34 women (17 CGM and 17 control group) who conceived during the 24-week planning pregnancy trial did not differ (figure 2, appendix p 5).

**Table 2:** Glycaemic control of pregnancy trial participants based on available HbA_1c_ data

	**CGM**	**Control**	**p value**
Baseline	6·83% (0·67)	6·95% (0·66)	··
24 weeks' gestation	6·23% (0·53)	6·40% (0·68)	··
Change from baseline to 24 weeks	−0·67 (0·58)	−0·52 (0·55)	0·0374
34 weeks' gestation	6·35% (0·57)	6·53% (0·70)	··
Change from baseline to 34 weeks	−0·54 (0·62)	−0·35 (0·65)	0·0372
Achieved HbA_1c_≤6·5% (48 mmol/mol) at 34 weeks	63/95 (66%)	48/92 (52%)	0·0601

Data are mean percentage (SD). Assessed in 99 participants in the CGM group and 96 participants in the control group at baseline, and in 95 participants in the CGM group and 92 participants in the control group at baseline and 34 weeks' gestation. Percentage point changes are either cross-sectional on participants with data for baseline, week 24, and week 34 values, or summaries of change on participants with data at the relevant timepoints. p values are from linear regression (HbA_1c_) or logistic regression (HbA_1c_<6·5%) on available data, controlling for baseline HbA_1c_ and method of insulin delivery. CGM=continuous glucose monitoring. HbA_1c_=glycated haemoglobin.

We found no evidence of heterogeneity in the CGM treatment effect across countries and no heterogeneity across differing baseline HbA_1c_ levels or for differing methods of insulin delivery (appendix p 6). Adjustment for baseline covariates including maternal education, smoking, body-mass index, duration of diabetes, and episodes of severe hypoglycaemia during the 12 months before enrolment also did not change the treatment effect.

Although every effort was made to ensure complete HbA_1c_ datasets, this was not achieved because of missing samples (∼20%) owing to withdrawals, lost samples, or participants being unavailable (admissions or preterm delivery). For the primary outcome, the patterns of change were similar between analyses of imputed and available HbA_1c_ data (appendix p 6).

In the pregnancy trial, the women in the CGM group spent increased time in the recommended glucose control target range of 3·5–7·8 mmol/L and reduced time above target compared with those in the control group at 34 weeks' gestation (table 3). Women in the CGM group also had reduced glucose SD, lower mean amplitude of glucose excursion, and non-significantly reduced glucose coefficient of variation, suggesting less glycaemic variability (table 3). The improvement in glucose control was achieved without increased maternal hypoglycaemia, gestational weight gain, or total daily insulin dose, but with an increase in the rate of change in glucose concentrations. Apart from an increase in the rate of change in glucose concentrations, we found no between-group differences in the secondary glycaemic outcomes in the planning pregnancy trial (appendix p 8).

**Table 3:** Glycaemic and adverse outcomes of pregnancy trial participants

		**Baseline**		**34 weeks' gestation**		p value*
		CGM	Control	CGM	Control	
**Direct CGM measures**†						
Hours per week‡		158 (143–168)	150 (139–165)	159 (143–177)	156 (143-166)	··
Glucose		7·3 (1·2)	7·6 (1·1)	6·7 (0·9)	7·0 (1·1)	0·14
Time in target		52% (13)	52% (14)	68% (13)	61% (15)	0·0034
Time >7·8 mmol/L		39% (28–49)	40% (32–51)	27% (19–37)	32% (25–39)	0·0279
High blood glucose index		4·2 (2·3–6·2)	4·6 (2·8–6·7)	1·8 (1·1–2·8)	2·3 (1·5–3·4)	0·067
Time <3·5 mmol/L		8% (4–14)	6% (3–11)	3% (1–6)	4% (2–8)	0·10
Low blood glucose index		2·8 (1·6–4·6)	2·4 (1·5–3·6)	1·7 (1·1–2·8)	2·1 (1·4–2·8)	0·18
Hypoglycaemia§		0·8 (0·6–1·0)	0·7 (0·4–0·9)	0·5 (0·3–0·8)	0·5 (0·3–0·8)	0·73
Glucose variability measures						
	Coefficient of variation	42% (38–47)	42% (36–47)	32% (28–37)	34% (29–39)	0·058
	SD (mmol/L)	3·1 (2·6–3·6)	3·1 (2·6–3·8)	2·2 (1·8–2·5)	2·4 (2·0–2·8)	0·0359
	Mean amplitude of glucose excursion (mmol/L)	6·0 (5·1–7·1)	6·4 (5·5–7·8)	4·2 (3·5–4·9)	4·6 (3·9–6·0)	0·0455
	Rate of change mmol/L per h	2·15 (1·88–2·52)	2·17 (1·89–2·46)	2·02 (1·70–2·26)	1·63 (1·31–1·96)	<0·0001
**Severe hypoglycaemia**¶						
Number of women		7 (7%)	4 (4%)	11 (11%)	12 (12%)	1·0
Number of episodes‡		11	5	18	21	··
Diabetic ketoacidosis during study		··	··	2 (2%)	2 (2%)	1·0
Changed to insulin pump during study		··	··	1 (1%)	3 (3 %)	0·62
Total insulin dose (U/kg per day)		0·69 (0·25)	0·76 (0·31)	0·99 (0·41)	1·07 (0·42)	0·14

Values are mean (SD) and median (IQR) as appropriate.

* p value for between-group difference at 34 weeks' gestation.

† CGM data were obtained 1 week after completion of the 34 week visit using real-time sensors in the CGM group and masked sensors in the control group. Assessed in 107 participants in the CGM group and 107 participants in the control group at baseline, and in 77 participants in the CGM group and 77 participants in the control group at 34 weeks' gestation.

‡ Not study outcomes.

§ Hypoglycaemia events are defined as CGM levels <3·5 mmol/L for at least 20 min. Distinct events were counted only if separated by at least 30 min.

¶ Severe hypoglycaemia was defined as an episode requiring third-party assistance; assessed in 107 participants in the CGM group and 107 participants in the control group at baseline, and in 103 participants in the CGM group and 104 participants in the control group at 34 weeks' gestation.

Total incidence of severe hypoglycaemia episodes across pregnancy did not differ between groups (table 3). CGM measures showed comparable time spent below target and comparable biochemical hypoglycaemia events at 34 weeks' gestation (table 3). Overnight and daytime CGM measures displayed a similar pattern, with increased time in target, and decreased hyperglycaemia in women in the CGM group in both the overnight and daytime periods (appendix pp 9–10). The CGM measures were comparable in insulin pump and injection users (appendix pp 11–12).

In the pregnancy trial, we observed no between-group differences in hypertensive disorders, pre-eclampsia, caesarean delivery, gestational age, or preterm delivery (table 4). Between-group differences in time in target, hyperglycaemia, and glucose variability became apparant in late gestation in the pregnancy trial with no differences at either timepoint in the pregnancy planning trial (appendix pp 13–14).

**Table 4:** Obstetric and neonatal health outcomes of pregnancy trial participants

		**CGM**	**Control**	**p value**
**Maternal outcomes**				
Number assessed		100	102	··
Hypertensive disorders		18 (18%)	28 (27%)	0·13
	Worsening chronic	2 (2%)	4 (4%)	0·68
	Gestational	8 (8%)	9 (9%)	1·0
	Pre-eclampsia	9 (9%)	18 (18%)	0·10
Caesarean section		63 (63%)	74 (73%)	0·18
Maternal weight gain (kg)*				
	Entry to 34 weeks	13·1 (9·9–16·6)	13·7 (10·9–17·6)	0·22
	From 16 to 34 weeks	8·9 (6·6–11·3)	9·7 (8·3–11·8)	0·09
Maternal length of stay (days)		3·5 (2·6–5·3)	4·2 (2·9–6·8)	0·10
**Neonatal outcomes**				
Number assessed		105	106	··
Pregnancy loss <20 weeks		5 (5%)	4 (4%)	1·0
Stillbirth		0	1	··
Termination		0	1	··
Congenital anomaly†		2	3	··
Preterm births				
	Number assessed	100	102	··
	Preterm <37 weeks	38 (38%)	43 (42%)	0·57
	Early preterm <34 weeks	5 (5%)	11 (11%)	0·19
Gestational age at delivery‡		37·4 (36·7–38·1)	37·3 (36·0–38·0)	0·50
Birthweight				
	Number assessed	100	100	··
	Birthweight (g)	3545·4 (649·0)	3582· (777·0)	0·37
	Median customised centile§	92 (68–99)	96 (84–100)	0·0489
	Small for gestational age (<tenth centile)	2 (2 %)	2 (2%)	1·0
	Large for gestational age (>90th centile)	53 (53%)	69 (69%)	0·0210
	Extremely large for gestational age (>97·7th centile)	36 (36%)	44 (44%)	0·31
Macrosomia (≥4000 g)		23 (23%)	27 (27%)	0·62
Neonatal complications				
	Number assessed	100	100	··
	Birth injury	1 (1%)	0	1·0
	Shoulder dystocia	1 (1%)	0	1·0
	Neonatal hypoglycaemia requiring intravenous dextrose	15 (15%)	28 (28%)	0·0250
	Hyperbilirubinaemia	25 (25%)	31 (31%)	0·43
	Respiratory distress	9 (9%)	9 (9%)	1·0
	High-level neonatal care (NICU) >24 h	27 (27%)	43 (43%)	0·0157
Infant length of hospital stay		3·1 (2·1–5·7)	4·0 (2·4–7·0)	0·0091
Composite neonatal outcome¶		45 (42·9%)	56 (52·8%)	0·17

Values are mean (SD) and median (IQR) as appropriate. CGM=continuous glucose monitoring. NICU=neonatal intensive care unit.

* Entry weight was self-reported or recorded pre-pregnancy weight, or both. The weight from 16 to 34 weeks was measured.

† Congenital anomalies were aortic stenosis and hypospadias grade 1 (CGM group) and hypoplastic right heart syndrome (termination of pregnancy), aberrant right subclavian artery, and bilateral hydronephrosis (control group).

‡ Gestational age at delivery was calculated only for the 100 pregnancies in the CGM group and the 101 pregnancies in the control group that were ongoing after 24 weeks' gestation.

§ Based on gestation-related optimal weight customised growth charts.

¶ Composite outcome comprises pregnancy loss (miscarriage, stillbirth, and neonatal death); birth injury; neonatal hypoglycaemia; hyperbilirubinaemia; respiratory distress; and high-level neonatal care for more than 24 h.

**Figure 3:**
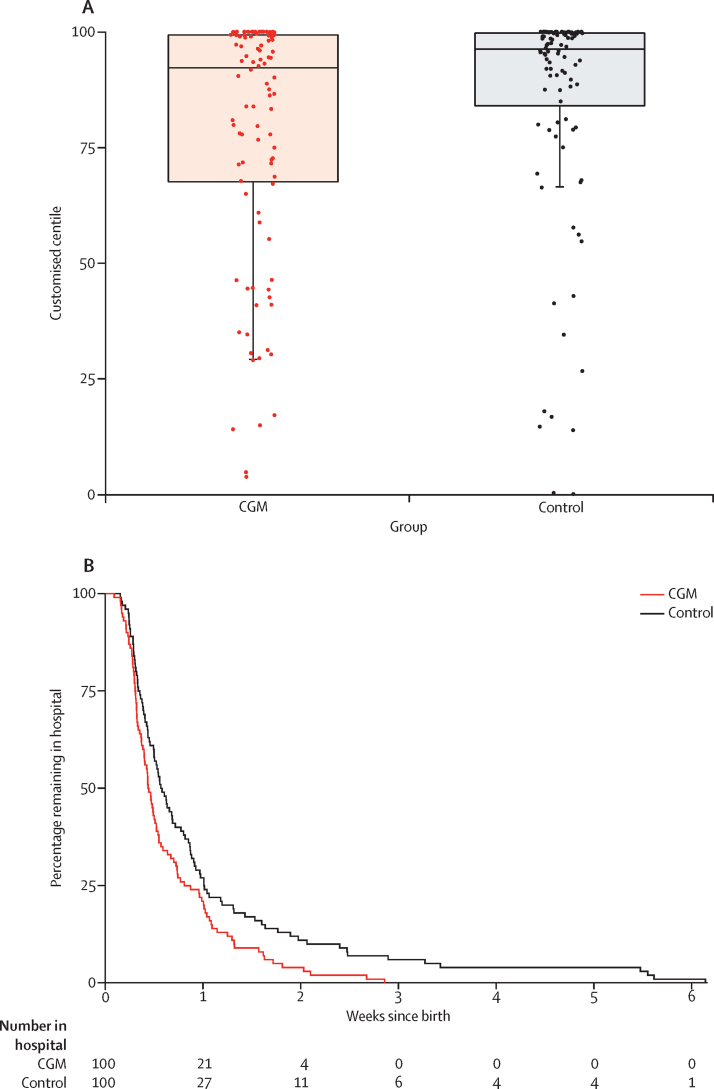
Neonatal outcomes of pregnancy trial participants (A) Neonatal birthweight centiles are shown with box plots. The horizontal line in the middle of each box represents the median, and the lower and upper boundaries of the box represent the 25th and 75th percentiles, respectively. Whiskers are drawn to the smallest value that is within 1·5 × IQR below the 25th percentile. Values outside of the whiskers are drawn individually. These data are based on customised growth charts (gestation-related optimal weight) that adjust infant birthweight for maternal parity, ethnicity, height, and weight, and for infant sex and gestational age.^20^ (B) The Kaplan-Meier plot shows infants' length of hospital stay from delivery until hospital discharge.

For neonatal outcomes, we found a decreased proportion of large for gestational age (odds ratio 0·51, 95% CI 0·28–0·90; p=0·0210) in the infants of mothers randomly assigned to CGM (figure 3, table 4). Despite substantial variation in birthweight across countries, we did not detect any differences in the effect of CGM on the proportion of infants large for gestational age across countries (likelihood ratio for heterogeneity 0·14; p=0·99; appendix p 25), although the trial was not powered to detect anything less than substantial heterogeneity.

Infants of mothers randomised to CGM experienced fewer neonatal intensive care admissions lasting more than 24 h (odds ratio 0·48, 95% CI 0·26–0·86; p=0·0157), fewer incidences of neonatal hypoglycaemia requiring treatment with intravenous dextrose (0·45, 0·22–0·89; p=0·0250), and a reduced total length of hospital stay (p=0·0091) than did infants of control participants (figure 3, table 4). Therefore, six women would need to be treated to prevent one neonatal intensive care unit admission over 24 h and one large-for-gestational-age infant, and eight women to prevent one neonatal hypoglycaemia event. We found no differences in the composite fetal outcome, cord blood C-peptide levels, and neonatal anthropometric measurements (table 4, appendix pp 15–16).

The obstetric and neonatal outcomes of the 34 women who conceived during the planning pregnancy trial are shown separately (appendix p 17). Their small numbers preclude further statistical analyses.

We found no between-group differences in any of the patient-reported outcome measures (appendix pp 18–19). Significant group × time interaction effects were obtained for the BGMSRQ total score (p=0·0431) and the HFS Behavior subscale (p=0·0347) during pregnancy. This result suggests that pregnant women in both groups became more satisfied over time, with satisfaction increasing slightly more in women in the CGM group. Hypoglycaemia avoidance behaviours decreased over time in women in the CGM group, but stayed constant over time in those in the control group (appendix p 18). CGM satisfaction scores indicated overall favourable ratings (mean scores 3·66–3·78 on a 4-point scale) in both trials.

More participants in the CGM groups experienced adverse events than in the control groups, with recognised CGM frustrations affecting more than 80% of women (table 5; appendix pp 20, 22). The most common adverse events in both groups were skin reactions, occurring in 49 (48%) of 103 CGM participants and eight (8%) of 104 control participants during pregnancy and in 23 (44%) of 52 CGM participants and five (9%) of 57 control participants in the planning pregnancy trial (appendix p 20). During pregnancy the proportion of antenatal hospital admissions was comparable between both groups (appendix p 21).

**Table 5:** Adverse events in pregnancy trial participants

	**CGM (n=107)**	**Control (n=107)**	**Odds ratio (OR) or rate ratio (RR; 95% CI)**	**p value**
Participants with adverse events	51 (48%)	43 (40%)	OR 1·2 (0·8–1·8)	0·35
Number of events	109	78	RR 1·4 (1·0–1·8)	0·041
Participants with serious adverse events	8 (7%)	5 (5%)	OR 1·6 (0·5–4·9)	0·41
Number of serious adverse events	8	7	RR 1·1 (0·4–3·1)	0·82

All randomised participants were included. The serious adverse events were gastrointestinal (nausea and vomiting; n=4), respiratory or related to ear, nose, and throat (n=2), obstetric (n=2), diabetic ketoacidosis (n=1), headache or migraine (n=1), cortisol deficiency (n=1), skin rash (n=1), urinary or genital (n=1), foot drop (neurological; n=1), and breast cancer (n=1). CGM=continuous glucose monitoring.

Few serious adverse events occurred (15 in the pregnancy group and three in the planning pregnancy group), with no between-group differences in either the pregnancy or planning pregnancy trial (table 5, appendix p 22). The most common serious adverse events were gastrointestinal (nausea and vomiting in four participants during pregnancy and three participants planning pregnancy).

Because of the unexpected difference in unscheduled contacts, we did a post-hoc analysis adding the total number of unscheduled contacts that were not purely sensor-related to the main ANCOVA model. This addition had minimal impact on the CGM treatment effect estimate which remained significant (p=0·0427, appendix p 7). Furthermore, the number of unscheduled contacts appeared unrelated to the change in HbA_1c_ (appendix p 23).

## Discussion

In this multicentre international, randomised controlled trial, women with type 1 diabetes randomised to CGM during early pregnancy had a small but significantly greater reduction in HbA_1c_ levels than did the control participants, accompanied by increased time in target, reduced hyperglycaemia, and less glycaemic variability at 34 weeks' gestation. In these women, we found reductions in the proportion of infants large for gestational age, neonatal hypoglycaemia, and admission to neonatal intensive care, and a 1-day shorter hospital stay among their infants. In women who were planning pregnancy, the point estimate of glycaemic change with CGM use was of a similar magnitude but with greater uncertainty, potentially due to smaller sample size or greater variation.

The numbers of pregnant women needed to treat with CGM to prevent one complication are six for both neonatal intensive care admission and large for gestational age, and eight for neonatal hypoglycaemia. Further health-economic analyses will be required to understand the costs of CGM and its implementation into antenatal care, which might be offset by the reduction in neonatal intensive care admissions and reduced hospital stay.

The mechanisms for the improved neonatal outcomes in mothers randomly assigned to CGM during early pregnancy are unclear. The reduction in large for gestational age was seen both in countries with higher (Canada and UK) and with lower (Italy and Spain) prevalence. The clinically small improvement in HbA_1c_ seems unlikely to be the sole explanation for the observed reductions in neonatal complications. We chose HbA_1c_ as the primary outcome because of its strong clinical validity, but it has well established limitations during pregnancy when HbA_1c_ levels are influenced by gestational changes in red cell turnover, anaemia, and iron supplementation.^29^, ^30^, ^31^ Pregnant CGM users spent 68% of their time within the recommended glycaemic control target range compared with 61% for their control counterparts, translating to an additional 1·7 h/day in target. Pregnant CGM users also spent 27% of their time above 7·8 mmol/L compared with 32% for control participants, equivalent to approximately 1 h less per day spent hyperglycaemic. These direct CGM measures of day-to-day exposure to maternal hyperglycaemia might be more relevant for neonatal outcomes than are surrogate markers such as HbA_1c_.

In the authors' clinical experience, women are uncomfortable changing treatment modality during early pregnancy, and thus starting CGM in women planning pregnancy seemed logical. The direct CGM measures suggest a lower mean glucose and 5% higher time in target (1·2 h/day) but these were not significant (appendix p 8). Longer periods of CGM recordings might have increased the statistical power of the study. We found a similar between-group difference in HbA_1c_ levels, but with wide confidence intervals, either due to smaller sample size or more variation in the treatment effect. The motivation of participants planning pregnancy for optimising glucose control might have also been more variable than the motivation of pregnant participants, although 50% of CGM participants and 40% of control group participants in the planning pregnancy trial did achieve their glycaemic control target.

During pregnancy, CGM users had slightly more frequent scheduled and notably more unscheduled contacts. Problems encountered with CGM use were commonplace, with more than 80% of women reporting frustrations of CGM use, such as connectivity issues, alarms, and calibration errors, and almost 50% experiencing skin reactions, such as bleeding, erythema, and discomfort. The increased contacts for sensor-related diabetes management issues might also reflect the heightened anxiety of pregnant women seeing the 288 daily glucose values in real time, and the initial support required to incorporate CGM data into their diabetes routines. The increased rate of change of glucose concentrations most likely reflects CGM users' actions taken in response to out-of-range glucose levels. Post-hoc analysis showed that adjusting for additional contacts did not affect treatment effect and was unrelated to changes in HbA_1c_. Furthermore, contact for non-sensor related issues did not differ between groups.

It is difficult to make direct comparison with the smaller previous studies that included pregnant women with both type 1 and type 2 diabetes. We found a greater effect on neonatal outcomes, which is probably due to our larger sample size (compared with 46 and 119 type 1 diabetes pregnancies in UK and Danish trials^18^, ^19^), advances in sensor technology, and improved CGM compliance. The UK study^18^ of intermittent masked CGM achieved a larger between-group difference in maternal HbA_1c_ levels, but with less effect on large for gestational age. The direct CGM measures suggested that although women achieved lower mean HbA_1c_ (5·8% or 40 mmol/mol), they spent only 56% of time in target, 33% above target, and 13% below target.^15^ The Danish trial^19^ used an earlier CGM technology intermittently with one real-time CGM on five occasions for the intervention group.^19^ They reported comparable HbA_1c_ and glucose control to the UK study (58% in target, 28% above, and 14% below target based on capillary glucose levels).

Our pregnant CGM users spent substantially higher time in target (68%) compared with previous studies, with approximately 10% or 2·4 h/day less hypoglycaemia. Of note, 19 (16%) of 119 Danish women had 59 severe hypoglycaemia episodes^19^ compared with 23 (11%) of 207 women with 39 severe hypoglycaemia episodes in our study. This suggests that, over the past decade, the burden of severe hypoglycaemia has substantially reduced. Only a minority of pregnant women (<20%) were using pumps with insulin suspend features, so this alone does not explain the reduced incidence of maternal hypoglycaemia. However, exposure to hyperglycaemia—the so-called prandial problem—persists. Post-prandial hyperglycaemia was also evident in our recent closed-loop study in pregnant women with type 1 diabetes,^32^ which achieved comparable time in target to the CGM users in the present study. These results indicate that additional strategies might be required to minimise post-prandial excursions, such as faster acting insulins or adjunctive therapies. Automated insulin delivery options might also give women more confidence to administer larger pre-meal boluses, especially for the evening meal, with less fear of nocturnal hypoglycaemia. Notably, although women in the CGM group showed a greater decrease in hypoglycaemia avoidance behaviour than did women in the control group, they did not have less worry, suggesting that automated insulin delivery might be more effective for reduction in maternal fear of hypoglycaemia.

Compared with the landmark Juvenile Diabetes Research Foundation study^17^ in which 90% of adults were using pump therapy, the CGM group in our study included more than 50% women on multiple daily injections.^17^ These women did just as well in terms of their treatment response and had lower absolute HbA_1c_ levels compared with women using insulin pump therapy. The finding that the treatment effect of CGM is comparable between pump and injection users is very important for widening access to technology. This result is consistent with recent CGM studies outside of pregnancy which focused specifically on multiple injection users.^33^, ^34^ These studies using more recent CGM technology achieved sensor compliance approaching 90% use, with another trial^35^ of a newer flash CGM device showing that users in the CGM group accessed their glucose levels 15 times per day compared with 5·6 capillary glucose tests.^35^

We note that even with the improvement obtained with CGM use, pregnancy outcomes remained suboptimal, with a high proportion of infants large for gestational age and high levels of neonatal morbidity. The UK pregnancy in diabetes audit, which used the same customised birthweight charts, reported a lower proportion of infants large for gestational age (46%) despite higher maternal HbA_1c_ levels in early and late pregnancy and fewer women achieving target HbA_1c_ levels (40% compared with 66% of pregnant CGM users).^9^ These results suggest that, in addition to glucose, other nutrients might also be important. We also note that pregnant women in our study were in the overweight range and weight gain was higher than what is recommended by the Institute of Medicine.^36^ In addition to hyperglycaemia, these issues will need to be addressed to improve pregnancy outcomes in women with type 1 diabetes.

Our trial has several strengths. First, the sample size for the pregnancy trial was large enough to provide statistical power for a range of clinically relevant maternal and neonatal outcomes. Second, our trial has a robust randomised controlled design with prior publication of the clinical study protocol, CGM treatment algorithms, prespecified statistical analyses, central laboratory HbA_1c_ assays, and direct CGM measures of glycaemia. Third, it has high external validity, including 31 antenatal clinics with variable diabetes technology experience and providing results that are applicable to pregnant women using insulin pumps or multiple daily injections. The data for maternal and neonatal health outcomes are more than 99% complete.

However, our trial also has some limitations. The planning pregnancy trial did not have sufficient power to detect the magnitude of differences that were significant in the pregnancy trial. Although every effort was made to ensure complete HbA_1c_ and CGM datasets, this was not achieved because of missing or lost samples, withdrawals, and pregnancy losses or delivery before 34 weeks. However, the percentage of missing data (∼20%) fell within the prespecified power calculation assumptions and the patterns of change were similar in analyses of imputed and of available HbA_1c_ data. Women entering the trial had to demonstrate willingness to use CGM before randomisation. Unfortunately, we do not have data on the frequency of capillary glucose monitoring and its relationship to glucose control or on the use of insulin suspension. We observed more unscheduled contacts for women in the CGM group than in the control group. We also acknowledge that there are potential differences between the CGM data collected using real-time sensors in the CGM group and masked sensors in the control group, and a large number of statistical tests were conducted without adjustment for multiple testing.

To our knowledge, this study is the first to show an effect of continuous glucose monitoring on health outcomes other than glycaemic outcomes, and with substantial reductions in neonatal complications attributed to maternal hyperglycaemia. The results were consistent across 31 international study sites and comparable for women using insulin pumps or multiple daily injections, regardless of baseline glucose control. Our data indicate a role for offering CGM to all pregnant women with type 1 diabetes using intensive insulin therapy in the first trimester.

This online publication has been corrected. The corrected version first appeared at thelancet.com on October 20, 2017
