# Department of Error

**DOI:** 10.1016/S0140-6736(17)32712-5

**Published:** 2017-11-25

*Feig DS, Donovan LE, Corcoy R, et al. Continuous glucose monitoring in pregnant women with type 1 diabetes (CONCEPTT): a multicentre international randomised controlled trial.* Lancet *2017; **390:** 2347–59*—This Article should have been published under a Creative Commons CC BY 4.0 open-access license. In the author list, Elizabeth Asztalos was spelled incorrectly. These corrections have been made to the online version as of Oct 20, 2017, and the printed Article is correct.

