# Supplementary material for: Continuous glucose monitoring in pregnant women with type 1 diabetes (CONCEPTT): a multicentre international randomised controlled trial
Source: Lancet. 2017 Nov 25;390(10110):2347–59. doi: 10.1016/S0140-6736(17)32400-5 (PMC5713979; doi:10.1016/S0140-6736(17)32400-5)

THE LANCET

Supplementary appendix

This appendix formed part of the original submission and has been peer reviewed. We post it as supplied by the authors.

Supplement to: Feig DS, Donovan LE, Corcoy R, et al, on behalf of the CONCEPTT Collaborative Group. Continuous glucose monitoring in pregnant women with type 1 diabetes (CONCEPTT): a multicentre international randomised controlled trial. *Lancet* 2017; published online Sept 15. http://dx.doi.org/10.1016/S0140-6736(17)32400-5.

Table of Contents

CONCEPTT Collaborative Group (listed according to recruitment numbers): 2

Data safety monitoring board 2

Table S1: Study visits 3

Table S2: Continuous glucose monitoring (CGM) compliance 4

- S2a: CGM compliance of participants in pregnancy trial 4
- S2b: Hours of CGM usage during the pregnancy trial 4
- S2c: CGM compliance of participants in pregnancy planning trial 4
- S2d: Hours of CGM usage during the pregnancy planning trial 4

Table S3: Glycaemic outcomes of participants who conceived during the 24 week planning pregnancy trial 5

Table S4: Sensitivity analyses of primary outcome 6

- S4a: Adjustment for potentially unbalanced maternal variables 6
- S4b: Primary outcome results for available data and from multiple imputation 6
- S4c: Adjustment for additional contacts in the pregnant group 7

Table S5: Glycaemic and adverse outcomes in the planning pregnancy trial 8

Table S6: Night-time (23.00-07.00hr) glucose measures 9

Table S7: Daytime (07.00-23.00hr) glucose measures 10

Table S8: Continuous glucose measures in insulin pump users 11

Table S9: Continuous glucose measures in Multiple Daily Injection (MDI) users 12

Table S10: CGM measures at baseline 24 and at 34 weeks in pregnancy trial 13

Table S11: CGM measures at baseline, 12 and 24 weeks in pregnancy planning trial 14

Table S12: Additional neonatal outcomes 15

Table S13: Neonatal anthropometric measures 16

Table S14: Obstetric and neonatal outcomes of pregnancy planning participants who conceived during the 24-week trial 17

Table S15: Summary of the Patient Reported Outcome Measures in the Pregnancy Trial 18

Table S16: Summary of the Patient Reported Outcome Measures in the Pregnancy Planning Trial 19

Table S17: CGM problems and skin reactions 20

Table S18: Hospital admissions in pregnant women 21

Table S19: Adverse Events (occurrences) 22

- S19a: Serious Adverse Events (occurrences) 22

Figure S1: Change in HbA1c in relation to unscheduled antenatal visits 23

Figure S2: Neonatal birthweight percentiles according to country 24

Figure S3: Neonatal Large for Gestational Age (LGA) rates according to country 25

CONCEPTT Collaborative Group (listed according to recruitment numbers):

Cambridge University Hospitals NHS Foundation Trust, Cambridge, UK: Helen Murphy, Jeannie Grisoni, Carolyn Byrne, Sandra Neoh, Katy Davenport, (43); Alberta Health Services, University of Calgary, Calgary, Canada: Lois Donovan, Claire Gougeon, Carolyn Oldford, Catherine Young (39); King's College Hospital, London, UK: Stephanie Amiel, Katharine Hunt, Louisa Green, Helen Rogers, Benedetta Rossi (29); Mount Sinai Hospital, Toronto, Canada: Denice Feig, Barbara Cleave, Michelle Strom (22); Hospital de la Santa Creu i Sant Pau, Barcelona, Spain and CIBER-BBN, Zaragoza, Spain: Rosa Corcoy, Alberto de Leiva, Juan María Adelantado, Ana Isabel Chico, Diana Tundidor (22); The Ottawa Hospital General Campus, Ottawa, Canada: Erin Keely, Janine Malcolm, Kathy Henry (15); Ipswich Hospital NHS Trust, Ipswich, UK: Damian Morris, Gerry Rayman, Duncan Fowler, Susan Mitchell, Josephine Rosier (13); Norfolk and Norwich University Hospital, Norwich, UK: Rosemary Temple, Jeremy Turner, Gioia Canciani, Niranjala Hewapathirana, Leanne Piper (13); St. Joseph's Health Centre, London, Canada: Ruth McManus, Anne Kudirka, Margaret Watson (13); Niguarda ca' Granda Hospital, Milano, Italy: Matteo Bonomo, Basilio Pintaudi, Federico Bertuzzi, Giuseppina Daniela Corica, Elena Mion (12); Sunnybrook Health Sciences Centre, Toronto, Canada: Julia Lowe, Ilana Halperin, Anna Rogowsky, Sapida Adib (11); Glasgow Royal Infirmary, Glasgow, UK: Robert Lindsay, David Carty, Isobel Crawford, Fiona Mackenzie, Therese McSorley (10); McMaster University, Hamilton, Canada: John Booth, Natalia McInnes, Ada Smith, Irene Stanton, Tracy Tazzeo (8); Centre hospitalier universitaire de Québec, Quebec City, Canada: John Weisnagel (6); Queen's Medical Centre, Nottingham, UK: Peter Mansell, Nia Jones, Gayna Babington, Dawn Spick (6); Royal Victoria Infirmary, Newcastle Upon Tyne, Newcastle, UK: Malcolm MacDougall, Sharon Chilton, Terri Cutts, Michelle Perkins (6); Leeds Teaching Hospitals NHS Trust, Leeds, UK: Eleanor Scott, Del Endersby (6); Royal Infirmary of Edinburgh, Edinburgh, UK: Anna Dover, Frances Dougherty, Susan Johnston (6); Sheffield Teaching Hospitals NHS Foundation Trust, Sheffield, UK: Simon Heller, Peter Novodorsky, Sue Hudson, Chloe Nisbet (6); Izaak Walton Killam Health Sciences Centre (IWK), Halifax, Canada: Thomas Ransom, Jill Coolen, Darlene Baxendale (5); University Hospital Southampton NHS Foundation Trust, Southampton, UK: Richard Holt, Jane Forbes, Nicki Martin, Fiona Walbridge (6); Galway University Hospitals, Galway, Ireland: Fidelma Dunne, Sharon Conway, Aoife Egan, Collette Kirwin (4); Central Manchester University Hospitals NHS Foundation Trust, Manchester, UK: Michael Maresh, Gretta Kearney, Juliet Morris, Susan Quinn (4); South Tees Hospitals, NHS Foundation Trust, Middlesbrough, UK: Rudy Bilous, Rasha Mukhtar (4); Centre de Recherche du Centre Hospitalier de Université de Montréal (CR-CHUM), Montreal, Canada: Ariane Godbout, Sylvie Daigle (3); The Dudley Group NHS FT, Russells Hall Hospital, Dudley, UK: Alexandra Lubina Solomon, Margaret Jackson, Emma Paul, Julie Taylor (3); Kingston General Hospital, Queen's University, Kingston, Canada: Robyn Houlden, Adriana Breen (3); Guys and St Thomas' NHS Foundation Trust, London, UK: Anita Banerjee, Anna Brackenridge, Annette Briley, Anna Reid, Claire Singh (2); Royal University Hospital, Saskatoon, Canada: Jill Newstead-Angel, Janet Baxter (2); Grampian Diabetes Centre, Aberdeen, UK: Sam Philip, Martyna Chlost, Lynne Murray (2); William Sansum Diabetes Center, Santa Barbara, USA: Kristin Castorino, Lois Jovanovic, Donna Frase (2). Juvenile Diabetes Research Foundation (non-clinical collaborators): Olivia Lou and Marlon Pragnell.

Data safety monitoring board

Kevin Thorpe (University of Toronto, Toronto, Canada); Lelia Duley (Bradford Institute for Health Research, Bradford, UK); John Pickup (King's College London, London, UK); Michael Bracken and Richard Ehrenkranz (Yale University School of Medicine, New Haven, CT, USA).

Table S1: Study visits

	Pregnant			Planning pregnancy †		
	**CGM**	**Control**	**P-value ††**	**CGM**	**Control**	**P-value ††**
	N=103	N=104		N=52	N=57	
Total number of scheduled visits¶	739	706		260	292	
**Visits per participant***	**7.2±1.1**	**6.8±1.4**	**0.0171**	5.0±1.6	5.1±1.5	0.71
Total number of unscheduled contacts†	1530	1026		418	333	
Diabetes management	857	858		237	265	
	8.3±7.9	8.2±7.4	0.92	4.6±5.9	4.6±5.3	0.47
**CGM issues**	**213**	**8**		**109**	**3**	
	**2.1±2.8**	**0.1±0.3**	**<0.0001**	**2.1±2.5**	**0.05±0.3**	**<0.0001**
**Diabetes and CGM issues**	**269**	**25**		**30**	**10**	
	**2.6±5.3**	**0.2±0.7**	**<0.0001**	**0.6±1.2**	**0.2±0.8**	**0.02**
Other	191	135		42	55	
	1.8±3.5	1.3±2.2	0.31	0.8±1.2	1.0±1.2	0.62

¶ Participants who completed at least one study visit are included

*Values are means ±SD

†Unscheduled contacts are shown as the total number and the mean (SD) per participant. These included face-to-face visits as well as telephone or email contacts

††P-values for continuous variables are from Wilcoxon rank-sum tests

Table S2: Continuous glucose monitoring (CGM) compliance

S2a: CGM compliance of participants in pregnancy trial

	**Overall**	**≤24 Weeks**	**25-34 Weeks**
**Days per week**	N=108	N=108	N=108
Zero	8 (7%)	8 (7%)	16 (15%)
<1 day	7 (6%)	6 (6%)	7 (6%)
1-<2 days	6 (6%)	4 (4%)	1 (<1%)
2-<3 days	2 (2%)	3 (3%)	1 (<1%)
3-<4 days	4 (4%)	6 (6%)	2 (2%)
4-<5 days	6 (6%)	5 (5%)	6 (6%)
5-<6 days	16 (15%)	21 (19%)	14 (13%)
6-<7 days	48 (44%)	37 (34%)	33 (31%)
7 days	11 (10%)	18 (17%)	28 (26%)
median	6.1	6.0	6.5
(25th, 75th percentiles)	(4.0, 6.8)	(4.0, 6.7)	(3.9, 7.0)
<6 days	49 (45%)	53 (49%)	47 (44%)
≥6 days	59 (55%)	55 (51%)	61 (56%)

S2b: Hours of CGM usage during the pregnancy trial

	**Weeks <13**	**Weeks 13-17**	**Weeks 18-21**	**Weeks 22-25**	**Weeks 26-29**	**Weeks 30-34**
**Pregnant**	N=71*	N=108	N=108	N=108	N=108	N=108
Zero use (# of women)	12	15	19	14	18	22
Median hours per week	133	123	124	131	130	129
(25th, 75th percentiles)	(72, 155)	(61, 150)	(70, 147)	(68, 151)	(69, 148)	(57, 149)
[range]	[0, 167]	[0, 164]	[0, 164]	[0, 162]	[0, 164]	[0, 164]

Participants in the CGM group who discontinued the intervention or withdrew from the trial are considered as having zero use.

S2c: CGM compliance of participants in pregnancy planning trial

	**Overall**	**<=12 Weeks**	**13-24 Weeks**
**Days per week**	N=52*	N=52	N=43
Zero	1 (2%)	1 (2%)	3 (7%)
<1 day	0 (0%)	0 (0%)	3 (7%)
1-<2 days	3 (6%)	3 (6%)	1 (2%)
2-<3 days	1 (2%)	0 (0%)	1 (2%)
3-<4 days	3 (6%)	0 (0%)	1 (2%)
4-<5 days	4 (8%)	7 (13%)	6 (14%)
5-<6 days	10 (19%)	6 (12%)	5 (12%)
6-<7 days	19 (37%)	23 (44%)	8 (19%)
7 days	11 (21%)	12 (23%)	15 (35%)
median	6.2	6.7	6.3
(25th, 75th percentiles)	(5.2, 6.9)	(5.3, 6.9)	(4.1, 7.0)
<6 days	22 (42%)	17 (33%)	20 (47%)
≥6 days	30 (58%)	35 (67%)	23 (53%)

*53 participants were randomised but one woman conceived before randomisation. Women who become pregnant (n=17) are included up to the point they conceive.
Participants in the CGM group who discontinued the intervention or withdrew from the trial are considered as having zero use.

S2d: Hours of CGM usage during the pregnancy planning trial

	**Weeks 1-4**	**Weeks 5-8**	**Weeks 9-12**	**Weeks 13-16**	**Weeks 17-20**	**Weeks 21-24**
**Pregnant**	N=52	N=48	N=46	N=43	N=40	N=38
Zero use (# of women)	1	3	1	3	7	7
Median hours per week	139	138	135	129	123	118
(25th, 75th percentiles)	(116, 156)	(115, 155)	(105, 150)	(93, 152)	(66, 152)	(74, 148)
[range]	[0, 165]	[0, 163]	[0, 163]	[0, 163]	[0, 164]	[0, 160]

Table S3: Glycaemic outcomes of participants who conceived during the 24 week planning pregnancy trial

	CGM	Control
HbA1c levels◊		
At pregnancy confirmation	6.91±0.45	7.01±0.54
HbA1c at 24 weeks gestation	6.24 ±0.47	6.49±0.69
Change from pregnancy confirmation to 24 weeks gestation	-0.50±0.36	-0.58±0.56
HbA1c at 34 weeks gestation	6.36±0.42	6.63±0.68
Change from pregnancy confirmation to 34 weeks gestation	-0.39±0.32	-0.46±0.67
Achieving target HbA1c level*	6 (66.7%)	7 (46.7%)
	N=14	N=16
Severe hypoglycaemia‡	2 (14.3%)	2 (12.5%)
Diabetic ketoacidosis	1 (7.1%)	0
Changed from pump to injections	0	0
Changed from injections to pump	0	1
	N=10	N=15
Total insulin dose at 34 weeks (Unit/kg/day)	1.21±0.72	0.93±0.31

◊ At pregnancy confirmation, 24 and 34 weeks n=15, 9, 9 for CGM and n=16, 15, 15 control group participants for central lab HbA1c levels.

*The target levels for HbA1c were ≤7.0% (53mmol/mol) before pregnancy and ≤6.5% (48mmol/mol) during pregnancy.

‡ Severe hypoglycaemia is from randomisation to 36 weeks gestation.

Table S4: Sensitivity analyses of primary outcome

S4a: Adjustment for potentially unbalanced maternal variables

Characteristic	Estimate	95% CI	P-value
	Pregnant participants		
**Baseline HbA1c (per %)**	**0.50**	**0.36, 0.64**	**<0.0001**
BMI (per kg/m^2)	0.00	-0.02, 0.02	0.94
Post-secondary level education (vs. lower)	0.04	-0.16, 0.24	0.68
Smoking (ever vs. never)	0.03	-0.20, 0.27	0.77
Duration of diabetes (years)	0.01	-0.01, 0.02	0.33
Severe hypoglycaemia (vs. not)	-0.11	-0.43, 0.20	0.48
**Multiple daily injections (vs. pump)**	**-0.22**	**-0.39, -0.04**	**0.0145**
**CGM (vs control)**	**-0.18**	**-0.35, -0.01**	**0.0382**
	Pregnancy planning participants		
**Baseline HbA1c (per %)**	**0.67**	**0.47, 0.86**	**<0.0001**
BMI (per kg/m^2)	0.01	-0.02, 0.04	0.51
Post-secondary level education (vs. lower)	0.20	-0.17, 0.56	0.29
**Smoking (ever vs. never)**	**0.47**	**0.02, 0.93**	**0.0415**
Duration of diabetes (years)	0.01	-0.01, 0.02	0.40
**Severe hypoglycaemia (vs. not)**	**0.56**	**0.12, 1.01**	**0.0134**
Multiple daily injections (vs. pump)	0.10	-0.21, 0.40	0.53
CGM (vs control)	-0.13	-0.39, 0.12	0.31

Table S4a shows estimates, 95% confidence intervals and p-values from linear regression models fitted to the available data in each group. Final HbA1c (%) is the outcome; in addition to the study intervention, baseline HbA1c and insulin delivery system, variables were included in both models if there was any concern about random imbalance in these variables after inspection of their distributions across treatment arms.

S4b: Primary outcome results for available data and from multiple imputation

	**Pregnant Estimate (95% CI; p-value)**		**Planning Pregnancy Estimate (95% CI; p-value)**	
	Available data	Multiple Imputation	Available data	Multiple Imputation
Intercept	6.66 (6.51, 6.81; <0.0001)	6.63 (6.49, 6.77; <0.0001)	7.31 (7.11, 7.50; <0.0001)	7.29 (7.09, 7.48; < 0.0001)
Baseline HbA1c (centred at mean)	0.51 (0.37, 0.65; <0.0001)	0.50 (0.38, 0.63; <0.0001)	0.64 (0.45, 0.84; < 0.0001)	0.64 (0.45, 0.84; < 0.0001)
Multiple daily injections (vs. pump)	-0.23 (-0.4, -0.06; 0.007)	-0.17 (-0.33, -0.01; 0.0401)	0.13 (-0.18, 0.43; 0.42)	0.19 (-0.11, 0.49; 0.20)
CGM (vs control)	-0.18 (-0.34, -0.01; 0.0372)	-0.19 (-0.34, -0.03; 0.0207)	-0.18 (-0.45, 0.08; 0.17)	-0.17 (-0.43, 0.09; 0.20)

Table S4b shows the estimates, CIs and p-values from fitting the ANCOVA model to available data (no missing baseline or final HBA1c) and from pooling the ANCOVA results across 40 datasets with missing outcomes imputed using the mice procedure in R (with all HbA1c values ,age at entry, insulin delivery system and treatment assignment in the imputation scheme).

S4c: Adjustment for additional contacts in the pregnant group

Characteristic	Estimate	95% CI	P-value
**Baseline HbA1c (per %)**	**0.51**	**0.37, 0.65**	**<0.0001**
**Multiple daily injections (vs. pump)**	**-0.23**	**-0.40, -0.06**	**0.0078**
Number of non-CGM visits (/10 visits)	-0.01	-0.09, 0.08	0.8953
**CGM (vs control)**	**-0.17**	**-0.34, -0.01**	**0.0427**

Table S4c shows estimates from the linear regression mode for the primary outcome in the pregnant group, with CGM vs. control as the comparison of interest, and baseline HbA1c, insulin delivery system and the number of non-purely-CGM-related unscheduled contacts as covariates. The adjustment for post-randomisation differences in participant contacts is an exploratory post-hoc analysis and should be read with caution.

Table S5: Glycaemic and adverse outcomes in the planning pregnancy trial

Planning pregnancy participants					
	Baseline		Follow-up †		
	CGM	Control	CGM	Control	P-value
**HbA1c measures**	N=46	N=52	N=42	N=46	
Baseline to 12 weeks	7.57±0.77	7.57±0.58	7.30±0.70	7.34±0.61	
Change to 12 weeks			-0.35±0.72	-0.22±0.39	0.44
Baseline to 24 weeks	7.57±0.77	7.57±0.58	7.12±0.64	7.35±0.87	
Change to 24 weeks			-0.41±0.72	-0.23±0.65	0.17
Achieved HbA1c ≤7.0% (53mmol/mol) at 24 weeks			25 (52.1%)	21 (40.4%)	0.44
**Direct CGM measures‡**	N=53	N=57	N=39	N=52	
Hours per week	166 (149-172)	157 (142-166)	159 (142-168)	152 (139-165)	
Mean glucose ± SD	8.8±1.3	9.0±1.5	8.0±1.3	8.6±1.6	0.14
% Time in target	42±13	41±13	48±13	43±16	0.30
% Time > 7.8mmol/l	54 (45-62)	57 (44-65)	49 (40-57)	52 (39-65)	0.23
High BG index	7.5 (4.8-9.7)	7.0 (4.8-10.2)	5.9 (3.3-7.2)	6.7 (3.9-8.7)	0.18
% Time < 3.5mmol/l	3 (1-7)	2 (0-4)	4 (1-8)	3 (1-6)	0.15
Low BG index	1.3 (0.7-2.5)	1.0 (0.4-1.7)	1.8 (0.9-2.5)	1.3 (0.7-2.2)	0.41
Hypoglycaemia event¥	0.5 (0.1-0.7)	0.3 (0.1-0.6)	0.6 (0.2-0.8)	0.5 (0.1-0.7)	0.34
Glucose variability measures					
CV %	40 (36-45)	38 (33-45)	40 (35-44)	37 (33-42)	0.40
SD mmol/L	3.5 (3.0-4.4)	3.5 (2.7-4.1)	3.3 (2.5-3.7)	3.2 (2.7-3.7)	0.54
MAGE mmol/L	6.6 (5.7-7.9)	6.5 (5.6-7.9)	6.4 (4.8-7.5)	6.7 (5.6-7.4)	0.53
**Rate of change mmol/l/hour**	**2.18 (1.86-2.62)**	**2.14 (1.79-2.43)**	**2.82 (2.24-3.25)**	**2.13 (1.77-2.45)**	**<0.001**
**Other secondary outcomes**	N=53	N=57	N=52	N=57	
Severe Hypoglycaemia*****					
Number of women	3 (5.7%)	7 (12.3%)	7 (13.5%)	5 (8.8%)	0.54
Number of episodes	7	11	12	6	
Diabetic ketoacidosis	N/A	N/A	0	2 (3.5%)	0.50
Changed to insulin pump			0	2	0.50
Changed from injections to insulin pump#			0	2	0.50
	N=53	N=57	N=35	N=39	
Total insulin dose (U/kg/day)	0.61±0.19	0.61±0.16	0.61±0.17	0.65±0.16	0.31
**Adverse events**			N=53	N=57	
Number of women	N/A	N/A	12 (22.6%)	21 (36.8%)	
†Odds Ratio$CI_{95\%}$			0.5 (0.2-1.1)		0.10
Number of events			24	47	
**†RateRatio$CI_{95\%}$**			**0.6 (0.3-0.9)**		**0.03**
Number of women with serious adverse events			2 (3.8%)	1 (1.8%)	
†Odds Ratio$CI_{95\%}$			2.1 (0.2-23.9)		0.55
Number of serious adverse events			2	1	
†RateRatio$CI_{95\%}$			2.2 (0.2-25.3)		0.51

Values are means ±SD and median (interquartile range) as appropriate
‡Continuous glucose measures were obtained after completion of the follow up visits using real-time sensors in the CGM group and masked sensors in the control group
¥Hypoglycaemia events are defined as continuous glucose levels <3.5mmol/L for at least 20 minutes. Distinct events were counted only if separated by at least 30 minutes.

*Severe hypoglycaemia was defined as an episode requiring third party assistance. Prior to randomisation there were 7 episodes in 3 CGM women and 11 episodes in 7 control group women. After randomisation there were 12 further episodes in 7 CGM women and 6 episodes in 5 control group women

†All randomised participants are included. Logistic regression analyses were used to estimate the odds of occurrence of an adverse event with 95% CIs by intervention versus control group. Poisson regression was used to calculate the rate of occurrence 95% CIs over the study period (randomisation until 24 weeks). P-values are from these models, with baseline HbA1c group and method of insulin delivery as covariates. The Serious Adverse Events were gastrointestinal (nausea and vomiting) in women planning pregnancy (n=3).

#One participant planning pregnancy in the control group changed from insulin pump to multiple doses injection and back to insulin pump.

Table S6: Night-time (23.00-07.00hr) glucose measures

Pregnant participants				
	Baseline		Follow-up†	
	CGM	Control	CGM	Control
	N=107	N=107	N=77	N=77
Mean glucose mmol/l	6.9±1.5	7.2±1.4	6.3±0.9	6.4±1.2
% Time in target	51±16	53±16	72±15	65±17
% Time > 7.8mmol/l	31 (20-48)	37 (22-49)	19 (10-32)	24 (11-35)
% Time < 3.5mmol/l	9 (3-23)	9 (4-15)	3 (1-9)	7 (1-15)
Hypoglycaemia episodes¥	1.3 (0.5-1.8)	1.0 (0.5-1.6)	0.6 (0.4-1.2)	0.8 (0.4-1.3)
CV %	40 (33-47)	42 (36-49)	28 (26-36)	32 (26-39)
SD mmol/L	2.6 (2.1-3.4)	3.0 (2.3-3.9)	1.8 (1.5-2.3)	2.1 (1.6-2.5)
MAGE mmol/L	3.9 (2.8-4.8)	4.6 (3.5-5.7)	2.8 (2.4-3.5)	3.2 (2.5-4.0)

Pregnancy planning participants				
	Baseline		Follow-up†	
	CGM	Control	CGM	Control
	N=53	N=57	N=39	N=52
Mean glucose mmol/l	8.7±1.9	8.9±2.1	7.8±1.6	8.4±2.0
% Time in target	41±17	41±17	49±19	45±21
% Time > 7.8mmol/l	50 (40-66)	54 (38-68)	41 (32-59)	50 (35-64)
% Time < 3.5mmol/l	3 (0-8)	1 (0-8)	6 (1-9)	3 (0-8)
Hypoglycaemia episodes¥	0.4 (0.0-1.0)	0.4 (0.0-0.9)	0.5 (0.0-1.0)	0.4 (0.0-0.9)
CV %	41 (32-47)	38 (30-45)	39 (35-42)	36 (29-41)
SD mmol/L	3.6 (2.8-4.2)	3.3 (2.5-4.2)	3.1 (2.3-3.6)	3.0 (2.4-3.6)
MAGE mmol/L	4.6 (3.4-5.5)	4.0 (3.2-5.5)	4.4 (3.3-5.8)	4.3 (3.1-5.6)

*Plus-minus values are means ±SD

‡ Continuous glucose measures were obtained after completion of the baseline and follow up visits using real-time sensor in the CGM and an IPro2 masked sensor in the control group

†At follow-up (34 weeks gestation during pregnancy and 24 weeks pregnancy planning) CGM data were available for 77 pregnant and 39 participants planning pregnancy

¥ Hypoglycaemia episodes are defined as continuous glucose levels <3.5mmol/L for at least 20 minutes. Distinct episodes were counted only if separated by at least 30 minutes.

Table S7: Daytime (07.00-23.00hr) glucose measures

Pregnant participants

	**Baseline**		**Follow-up†**	
	**CGM**	**Control**	**CGM**	**Control**
	**N=107**	**N=107**	**N=77**	**N=77**
Mean glucose mmol/l	7.6±1.3	7.7±1.2	6.9±1.0	7.3±1.3
% Time in target	52±14	51±15	65±14	59±16
% Time > 7.8mmol/l	41 (29-52)	41 (32-54)	29 (20-39)	36 (28-45)
% Time < 3.5mmol/l	6 (3-11)	4 (1-8)	2 (1-5)	3 (1-5)
Hypoglycaemia episodes[¥]	1.0 (0.6-1.4)	0.8 (0.3-1.3)	0.5 (0.2-1.0)	0.5 (0.3-0.9)
CV %	42 (37-46)	39 (34-45)	31 (28-35)	33 (29-37)
SD mmol/L	3.0 (2.6-3.6)	3.1 (2.5-3.7)	2.2 (1.8-2.5)	2.3 (2.0-3.0)
MAGE mmol/L	5.8 (4.9-7.0)	6.1 (5.3-7.5)	4.1 (3.4-4.8)	4.5 (3.9-5.8)

Pregnancy planning participants

	**Baseline**		**Follow-up†**	
	**CGM**	**Control**	**CGM**	**Control**
	**N=53**	**N=57**	**N=39**	**N=52**
Mean glucose mmol/l	8.9±1.4	9.0±1.4	8.1±1.3	8.7±1.7
% Time in target	42±14	40±13	47±14	43±16
% Time > 7.8mmol/l	55 (47-60)	56 (47-65)	53 (39-59)	49 (42-68)
% Time < 3.5mmol/l	2 (0-5)	1 (0-4)	2 (1-7)	2 (0-5)
Hypoglycaemia episodes[¥]	0.4 (0.2-0.8)	0.4 (0.0-0.7)	0.6 (0.2-1.0)	0.5 (0.0-0.9)
CV %	38 (35-45)	37 (34-43)	37 (34-43)	36 (32-41)
SD mmol/L	3.5 (2.9-4.0)	3.5 (2.8-4.0)	3.1 (2.5-3.6)	3.1 (2.7-3.7)
MAGE mmol/L	6.5 (5.4-7.3)	6.4 (5.3-7.8)	6.0 (4.7-7.0)	6.4 (5.8-7.1)

[¥] Hypoglycaemia episodes are defined as continuous glucose levels <3.5mmol/L for at least 20 minutes. Distinct episodes were counted only if separated by at least 30 minutes.

†At follow-up (34 weeks gestation during pregnancy and 24 weeks pregnancy planning)

Table S8: Continuous glucose measures in insulin pump users

Pregnant participants (N=98)

	**Baseline**		**Follow-up†**	
	**CGM**	**Control**	**CGM**	**Control**
	**N=50**	**N=48**	**N=35**	**N=37**
Mean glucose mmol/l	7.3±1.2	7.4±1.2	6.7±1.0	7.0±0.9
% Time in target	53±12	54±14	66±13	62±14
% Time > 7.8mmol/l	39 (26-47)	39 (29-49)	27 (20-37)	32 (27-41)
% Time < 3.5mmol/l	8 (3-13)	6 (3-10)	3 (1-7)	4 (2-7)
Hypoglycaemia episodes[¥]	0.8 (0.6-1.0)	0.7 (0.5-0.9)	0.5 (0.3-0.8)	0.5 (0.4-0.7)
CV %	42 (37-47)	40 (36-46)	31 (28-37)	35 (29-40)
SD mmol/L	3.0 (2.5-3.4)	3.1 (2.5-3.6)	2.2 (1.8-2.5)	2.4 (2.0-3.0)
MAGE mmol/L	5.9 (5.0-7.0)	6.2 (5.4-7.5)	4.4 (3.5-4.8)	4.8 (3.9-6.1)

Pregnancy planning participants (N=81)

	**Baseline**		**Follow-up†**	
	**CGM**	**Control**	**CGM**	**Control**
	**N=39**	**N=42**	**N=29**	**N=38**
Mean glucose mmol/l	8.8±1.3	8.9±1.4	8.0±1.1	8.5±1.4
% Time in target	42±14	40±13	49±13	45±15
% Time > 7.8mmol/l	53 (45-63)	57 (50-65)	49 (40-55)	50 (38-67)
% Time < 3.5mmol/l	3 (0-7)	2 (0-4)	4 (2-8)	2 (0-5)
Hypoglycaemia episodes[¥]	0.5 (0.1-0.7)	0.3 (0.1-0.5)	0.6 (0.3-0.8)	0.4 (0.1-0.6)
CV %	40 (36-48)	38 (32-42)	41 (36-44)	35 (33-40)
SD mmol/L	3.4 (2.9-4.5)	3.3 (2.6-4.0)	3.3 (2.5-3.7)	3.0 (2.6-3.5)
MAGE mmol/L	6.3 (5.6-8.2)	6.4 (5.4-7.8)	6.4 (4.8-7.4)	6.5 (5.2-7.1)

[¥] Hypoglycaemia episodes are defined as continuous glucose levels <3.5mmol/L for at least 20 minutes. Distinct episodes were counted only if separated by at least 30 minutes.

†At follow-up (34 weeks gestation during pregnancy and 24 weeks pregnancy planning)

Table S9: Continuous glucose measures in Multiple Daily Injection (MDI) users

Pregnant participants (N=116)

	**Baseline**		**Follow-up†**	
	**CGM**	**Control**	**CGM**	**Control**
	**N=57**	**N=59**	**N=42**	**N=40**
Mean glucose mmol/l	7.3±1.2	7.7±1.0	6.7±0.8	7.0±1.3
% Time in target	50±13	50±13	69±13	61±17
% Time > 7.8mmol/l	39 (30-49)	41 (34-51)	26 (17-36)	31 (24-39)
% Time < 3.5mmol/l	8 (5-17)	6 (2-12)	3 (1-6)	5 (2-9)
Hypoglycaemia episodes[¥]	0.8 (0.6-1.0)	0.7 (0.3-0.9)	0.5 (0.3-0.8)	0.5 (0.3-0.8)
CV %	43 (39-48)	43 (36-49)	33 (28-37)	34 (29-38)
SD mmol/L	3.2 (2.7-3.6)	3.2 (2.7-3.9)	2.2 (1.8-2.5)	2.3 (2.0-2.8)
MAGE mmol/L	6.3 (5.2-7.1)	6.6 (5.5-8.2)	4.2 (3.6-5.3)	4.6 (3.9-5.7)

Pregnancy planning participants (N=29)

	**Baseline**		**Follow-up†**	
	**CGM**	**Control**	**CGM**	**Control**
	**N=14**	**N=15**	**N=10**	**N=14**
Mean glucose mmol/l	8.9±1.3	9.1±1.6	8.1±1.8	8.9±2.1
% Time in target	39±12	42±14	44±15	40±17
% Time > 7.8mmol/l	55 (49-62)	56 (41-66)	54 (43-62)	54 (46-61)
% Time < 3.5mmol/l	2 (1-8)	3 (0-7)	3 (1-7)	6 (3-9)
Hypoglycaemia episodes[¥]	0.5 (0.1-0.8)	0.4 (0.0-0.7)	0.5 (0.2-0.7)	0.6 (0.5-0.7)
CV %	39 (35-45)	41 (36-48)	36 (35-42)	41 (38-46)
SD mmol/L	3.7 (3.0-3.8)	4.0 (3.2-4.1)	3.1 (2.6-3.4)	3.6 (3.2-4.5)
MAGE mmol/L	7.1 (5.7-7.7)	7.0 (6.0-9.4)	6.4 (5.7-7.5)	7.4 (5.9-8.2)

[¥] Hypoglycaemic episodes are defined as continuous glucose levels <3.5mmol/L for at least 20 minutes. Distinct episodes were counted only if separated by at least 30 minutes

[†]At follow-up (34 weeks gestation during pregnancy and 24 weeks pregnancy planning)

Table S10: CGM measures at baseline 24 and at 34 weeks in pregnancy trial

	**Pregnant participants**							
	**Baseline**		**Week 24**			**Week 34**		
	**CGM**	**Control**	**CGM**	**Control**	**P-value**	**CGM**	**Control**	**P-value**[†]
	**N=107**	**N=107**	**N=90**	**N=90**		**N=77**	**N=77**	
Hours of CGM Data	158 (143, 168)	150 (139, 165)	168 (147, 182)	160 (144, 165)	-	159 (143, 177)	156 (143, 166)	-
Glucose Control								
Mean (mmol/L)	7.3 ± 1.2	7.6 ± 1.1	7.6 ± 1.2	7.8 ± 1.3	0.53	6.7 ± 0.9	7.0 ± 1.1	0.14
% CGM 3.5-7.8 mmol/L	52% ± 13%	52% ± 14%	53% ± 15%	50% ± 15%	0.14	**68% ± 13%**	**61% ± 15%**	**0.0034**
Hyperglycaemia								
% CGM >7.8 mmol/L	39% (28%, 49%)	40% (32%, 51%)	43% (29%, 54%)	45% (33%, 54%)	0.76	**27% (19, 37%)**	**32% (25, 39%)**	**0.0279**
AUC >7.8 mmol/L	20 (11, 29)	22 (13, 32)	17 (10, 30)	21 (11, 28)	0.47	8 (4, 13)	10 (7, 16)	0.087
% CGM >6.7 mmol/L	51% (40%, 61%)	53% (46%, 63%)	58% (44%, 70%)	60% (49%, 69%)	0.51	45% (34%, 57%)	48% (42%, 55%)	0.14
AUC >6.7 mmol/L	30 (18, 39)	31 (21, 44)	27 (17, 42)	31 (20, 40)	0.46	**15 (9, 21)**	**18 (13, 26)**	**0.0489**
High BG index	4.2 (2.3, 6.2)	4.6 (2.8, 6.7)	3.6 (2.2, 6.3)	4.4 (2.5, 5.9)	0.44	1.8 (1.1, 2.8)	2.3 (1.5, 3.4)	0.067
Hypoglycaemia								
% CGM <3.5 mmol/L	8% (4%, 14%)	6% (3%, 11%)	3% (1%, 6%)	4% (1%, 8%)	0.42	3% (1%, 6%)	4% (2%, 8%)	0.10
AUC <3.5 mmol/L	0.8 (0.3, 1.7)	0.5 (0.2, 1.3)	0.3 (0.1, 0.6)	0.4 (0.1, 0.8)	0.38	0.2 (0.1, 0.6)	0.2 (0.1, 0.9)	0.17
% CGM <2.8 mmol/L	2% (0%, 6%)	1% (0%, 4%)	0% (0%, 2%)	1% (0%, 3%)	0.32	0% (0%, 2%)	1% (0%, 3%)	0.44
AUC <2.8 mmol/L	0.1 (0.0, 0.4)	0.1 (0.0, 0.3)	0.0 (0.0, 0.1)	0.0 (0.0, 0.2)	0.45	0.0 (0.0, 0.1)	0.0 (0.0, 0.2)	0.57
Low BG index	2.8 (1.6, 4.6)	2.4 (1.5, 3.6)	1.5 (0.9, 2.4)	1.7 (0.9, 2.7)	0.42	1.7 (1.1, 2.8)	2.1 (1.4, 2.8)	0.18
Episodes per 24h [a]	0.8 (0.6, 1.0)	0.7 (0.4, 0.9)	0.5 (0.3, 0.8)	0.5 (0.3, 0.8)	0.96	0.5 (0.3, 0.8)	0.5 (0.3, 0.8)	0.73
Glucose variability								
CV	42% (38%, 47%)	42% (36%, 47%)	35% (31%, 39%)	36% (32%, 41%)	0.27	32% (28%, 37%)	34% (29%, 39%)	0.058
SD (mmol/L)	3.1 (2.6, 3.6)	3.1 (2.6, 3.8)	2.6 (2.3, 3.1)	2.8 (2.3, 3.3)	0.24	**2.2 (1.8, 2.5)**	**2.4 (2.0, 2.8)**	**0.0359**
MAGE (mmol/L)	6.0 (5.1, 7.1)	6.4 (5.5, 7.8)	5.5 (4.6, 6.2)	5.9 (4.6, 6.7)	0.23	**4.2 (3.5, 4.9)**	**4.6 (3.9, 6.0)**	**0.0455**
Absolute rate of change	0.65 (0.56, 0.76)	0.65 (0.57, 0.74)	**0.67 (0.58, 0.77)**	**0.55 (0.48, 0.66)**	**<0.0001**	**0.61 (0.51, 0.68)**	**0.49 (0.39, 0.59)**	**<0.0001**

[¥]Hypoglycaemic episode defined as CGM readings <3.5 mmol/L for at least 20 minutes. Distinct episodes must be separated by at least 30 minutes.

[†]P-value at 24 and 34 weeks adjusted for baseline value and insulin modality.

Table S11: CGM measures at baseline, 12 and 24 weeks in pregnancy planning trial

	Pregnancy planning participants							
	Baseline		Week 12			Follow-up†		
	CGM	Control	CGM	Control	P-value†	CGM	Control	P-value†
	N=53	N=57	N=39 [a]	N=46 [a]		N=39 [b]	N=52 [b]	
Hours of CGM Data	166 (149, 172)	157 (142, 166)	160 (133, 174)	157 (141, 165)	-	159 (142, 168)	152 (139, 165)	
Glucose Control								
Mean (mmol/L)	8.8 ± 1.3	9.0 ± 1.5	8.4 ± 1.4	9.0 ± 1.6	0.03	8.0 ± 1.3	8.6 ± 1.6	0.14
% CGM 3.5-7.8 mmol/L	42% ± 13%	41% ± 13%	43% ± 14%	40% ± 14%	0.16	48% ± 13%	43% ± 16%	0.30
Hyperglycaemia								
% CGM >7.8 mmol/L	54% (45%, 62%)	57% (44%, 65%)	55% (36%, 63%)	57% (47%, 65%)	0.17	49% (40%, 57%)	52% (39%, 65%)	0.23
AUC >7.8 mmol/L	36 (23, 47)	33 (23, 48)	31 (17, 40)	36 (25, 49)	0.006	28 (15, 34)	32 (18, 41)	0.20
% CGM >6.7 mmol/L	67% (56%, 75%)	69% (62%, 79%)	66% (53%, 73%)	69% (59%, 77%)	0.34	62% (51%, 71%)	65% (54%, 78%)	0.28
AUC >6.7 mmol/L	49 (33, 61)	46 (33, 61)	42 (24, 54)	49 (36, 64)	0.007	39 (25, 48)	42 (28, 56)	0.20
High BG index	7.5 (4.8, 9.7)	7.0 (4.8, 10.2)	6.5 (3.6, 8.5)	7.5 (5.3, 10.4)	0.005	5.9 (3.3, 7.2)	6.7 (3.9, 8.7)	0.18
Hypoglycaemia								
% CGM <3.5 mmol/L	3% (1%, 7%)	2% (0%, 4%)	4% (1%, 7%)	3% (1%, 7%)	0.82	4% (1%, 8%)	3% (1%, 6%)	0.15
AUC <3.5 mmol/L	0.2 (0.0, 0.8)	0.2 (0.0, 0.4)	0.3 (0.0, 0.8)	0.2 (0.0, 1.0)	0.93	0.3 (0.1, 0.8)	0.2 (0.0, 0.6)	0.27
% CGM <2.8 mmol/L	1% (0%, 3%)	0% (0%, 1%)	1% (0%, 3%)	0% (0%, 3%)	0.98	1% (0%, 3%)	0% (0%, 2%)	0.33
AUC <2.8 mmol/L	0.0 (0.0, 0.2)	0.0 (0.0, 0.1)	0.0 (0.0, 0.2)	0.0 (0.0, 0.2)	0.77	0.0 (0.0, 0.2)	0.0 (0.0, 0.1)	0.53
Low BG index	1.3 (0.7, 2.5)	1.0 (0.4, 1.7)	1.5 (0.6, 2.3)	1.5 (0.6, 2.4)	0.85	1.8 (0.9, 2.5)	1.3 (0.7, 2.2)	0.41
Episodes per 24h [c]	0.5 (0.1, 0.7)	0.3 (0.1, 0.6)	0.5 (0.2, 0.8)	0.4 (0.2, 0.8)	0.69	0.6 (0.2, 0.8)	0.5 (0.1, 0.7)	0.34
Glucose variability								
CV	40% (36%, 45%)	38% (33%, 45%)	38% (35%, 44%)	40% (35%, 44%)	0.24	40% (35%, 44%)	37% (33%, 42%)	0.40
SD (mmol/L)	3.5 (3.0, 4.4)	3.5 (2.7, 4.1)	3.3 (2.8, 3.7)	3.5 (3.2, 4.2)	0.004	3.3 (2.5, 3.7)	3.2 (2.7, 3.7)	0.54
MAGE (mmol/L)	6.6 (5.7, 7.9)	6.5 (5.6, 7.9)	6.4 (5.7, 7.2)	6.9 (5.8, 8.5)	0.03	6.4 (4.8, 7.5)	6.7 (5.6, 7.4)	0.53
Absolute rate of change	0.66 (0.56, 0.79)	0.64 (0.54, 0.73)	0.83 (0.70, 0.94)	0.70 (0.59, 0.77)	<0.0003	0.85 (0.67, 0.98)	0.64 (0.53, 0.73)	<0.0001

a – Excludes women who became pregnant prior to the 12 week visit.

b – Includes the final CGM data for women who became pregnant prior to the 24 week visit.

c – Hypoglycaemic episode defined as CGM readings <3.5 mmol/L for at least 20 minutes. Distinct episodes must be separated by at least 30 minutes.

† P-value adjusted for baseline value and insulin modality.

Table S12: Additional neonatal outcomes

	CGM	Control	P-value*
	**N=83**	**N=80**	
Cord Blood pH <7.0	0	2	0.24
	N=61	N=61	
Cord Blood C-peptide pmol/L median (IQR)	695 (497-1354)	887 (513-1701)	0.37
Cord C-peptide >566 pmol/L◊	40 (65.6%)	42 (68.9%)	0.85
Cord C-peptide >2725 pmol/L†	6	6	1.0
	N=100	N=100	
Infant length of hospital stay			
Total number of days for hospital admission	455	697	
Median (IQR)	**3.1 (2.1-5.7)**	**4.0 (2.4-7.0)**	**0.01**
Late preterm ≥34 or <37 wks	33 (33.0%)	32 (32.0%)	1.0
Total number of days for hospital admission	198	260	
Median (IQR)	4.9 (2.3-7.2)	6.1 (3.0-9.7)	
Early preterm <34 wks	5 (5.0%)	10 (10.0%)	0.28
Total # of days for hospital admission	54	218	
Median (IQR)	11.0 (9.2-11.3)	19.8 (11.5-34.8)	
	N=99	N=98	
Hospital re-admission following first discharge home	14 (14.1%)	8 (8.2%)	0.26
Total number of times baby was re-admitted to hospital	17	9	

*P-values for continuous variables are from Wilcoxon rank sum tests; p-values for dichotomous outcomes are from Fisher's exact tests. The p-value for infant length of hospital stay is from log-rank test stratified for baseline HbA1c and insulin delivery method

◊ Cord c-peptide >566 pmol/L is based on >90th percentile value (>1.7 ug/L) in the HAPO study

† Cord c-peptide >2725 pmol/L is based on >90th percentile value in the CONCEPTT study

Table S13: Neonatal anthropometric measures

	CGM	Control	P-value*
**Anthropometric Measures**	**N=85**	**N=75**	
Biceps (mm)	5.19±1.67	4.87±1.26	0.44
Missing	N=17	N=15	
Triceps (mm)	5.93±1.85	5.96±1.69	0.78
Missing	N=14	N=12	
Subscapular (mm)	5.64±1.57	5.96±1.81	0.36
Missing	N=14	N=12	
Supraliliac (Flank) (mm)	5.04±1.80	5.43±1.95	0.24
Missing	N=16	N=13	
Sum of 4 skin folds (triceps, subscapular, biceps, flank)	21.9±5.9	22.1±5.6	0.67
Missing	N=17	N=15	
Sum of 4 skin folds >90th percentile	7 (10.3%)	6 (10.0%)	1.0
Sum of 3 skin folds (triceps, biceps, sub scapular)	16.8±4.5	16.7±4.0	0.84
Missing	N=17	N=15	
Sum of 3 skin folds >90th percentile	8 (11.8%)	5 (8.3%)	0.57
Head Circumference (cm)	34.3±2.1	34.5±1.7	0.76
Chest circumference (cm)	34.7±3.2	34.6±2.5	0.41
Missing	N=12	N=6	
Abdominal Circumference (cm)	34.2±3.1	34.2±2.9	0.70
Missing	N=11	N=7	
Left upper-arm circumference (cm)	11.4±1.3	11.7±1.4	0.12
Missing	N=13	N=7	
Crown-heel length (cm)	50.0±4.1	50.3±3.4	0.83
Missing	N=4	N=2	
Crown-rump length (cm)	33.4±4.5	34.3±4.4	0.16
Missing	N=12	N=8	
Head: Abdominal circumference Ratio	1.0±0.1	1.0±0.1	0.69
Missing	N=11	N=7	
Neonatal fat mass§	15.2±4.6	15.9±4.1	0.44
Missing	16	13	

Values are mean ± SD unless otherwise specified. P-values for continuous variables are from Wilcoxon rank sum tests; p-values for dichotomous outcomes are from Fisher's exact tests.

§ Neonatal fat mass was calculated using the mathematical model proposed by Catalano et al., which includes birth weight, length and flank skin-fold. Fat mass =0.39055 (birth weight) + 0.0453 (flank skin-fold) – 0.03237 (length) + 0.54657.

Table S14: Obstetric and neonatal outcomes of pregnancy planning participants who conceived during the 24-week trial

	CGM	Control
**Maternal outcomes**	**N=10**	**N=15**
Hypertensive disorders	1	5
Preeclampsia	0	1
Caesarean section	7	11
Maternal weight gain (kg)		
From entry to 34 weeks gestation median (IQR)	10.4 (7.3-13.9)	13.4 (9.9-16.2)
From 16 to 34 weeks gestation median (IQR)	7.7 (7.0-8.6)	10.5 (7.0-12.1)
**Neonatal outcomes**	N=14	N=17
Pregnancy Loss <20 weeks	4 (28.6%)	2 (11.8%)
Stillbirth	0	0
Termination	0	0
Congenital anomaly	0	0
	N=10	N=15
Gestational age at delivery weeks median (IQR)	37.0 (35.8-37.4)	37.6 (36.9-38.0)
Preterm birth	5	4
Early preterm <34 weeks	0	0
Birth weight (g) (live births) (mean±SD)	3544.2±582.9	3871.5±620.4
Customised centiles median (IQR)	94.0 (82.6-99.6)	97.9 (89.3-100.0)
SGA <10th centile	0	0
LGA >90th centile	6	11
LGA > 97.7th centile	4	9
≥4000g	2	7
Birth injury	0	0
Shoulder dystocia	0	0
Neonatal hypoglycaemia	7	7
Hyperbilirubinaemia	3	3
Respiratory Distress	0	1
High level neonatal care (NICU)	7	6
NICU length of stay > 24 hrs	5.3 (4.2-10.0)	3.0 (2.8-6.3)
Cord Blood pH <7.0	0	0
Composite fetal outcome	11 (78.6%) n=14	12 (70.6%) n=17
Cord Blood C-peptide pmol/L Median (IQR)	914.0 (619.0-1327.5) n=7	556.0 (490.0-1119.5) n=11
Cord C-peptide >566 pmol/L$^{◊}$	5	5
Cord C-peptide >2725 pmol/L)†	1	1
Sum of 4 skin folds Median (IQR)	18.9 (17.7-26.4) n=5	21.5 (19.6-23.4) n=11

◊ Cord c-peptide >566 pmol/L is based on >90th percentile in the HAPO study

† Cord c-peptide >2725 pmol/L is based on >90th percentile in CONCEPTT

Table S15: Summary of the Patient Reported Outcome Measures in the Pregnancy Trial

The group by time interaction refers to between-group differences between baseline (approximately 8-9 weeks gestation) and 34 weeks gestation

**QUESTIONNAIRE (Subscale)**		**Baseline Mean (SD)**	**34 Week Mean (SD)**	**Group x Time Interaction p**
**BGMSRQ Total**	CGM	89.5 (13.2)	98.2 (12.4)	**0.0431**
	Control	89.9 (16.1)	93.9 (14.2)	
**BGMSRQ Satisfaction**	CGM	34.3 (6.4)	35.9 (6.3)	NS
	Control	34.9 (7.5)	36.3 (6.5)	
**BGMSRQ Impact**	CGM	31.8 (7.0)	36.9 (6.6)	NS
	Control	31.5 (7.8)	32.1 (7.0)	
**BGMSRQ Obstruction**	CGM	25.2 (3.7)	23.5 (4.4)	NS
	Control	25.8 (3.4)	25.4 (4.5	
**HFS Total**	CGM	39.3 (21.8)	35.7 (20.4)	NS
	Control	36.6 (19.4)	33.9 (20.1)	
**HFS Behaviour**	CGM	16.5 (8.8)	16.4 (8.0)	**0.0347**
	Control	15.9 (7.2)	15.4 (7.4)	
**HFS Worry**	CGM	23.0 (15.2)	19.3 (14.5)	.078
	Control	20.8 (14.1)	18.5 (13.9)	
**PAID Total**	CGM	22.4 (15.7)	17.2 (13.7)	NS
	Control	17.7 (14.1)	16.4 (15.1)	
**SHORT FORM 12 Total**	CGM	46.6 (5.0)	41.7 (6.9)	NS
	Control	46.7 (6.1)	42.4 (6.5)	
**CGM-SAT Total**	CGM	-	161.2 (25.9)	N/A

Table S16: Summary of the Patient Reported Outcome Measures in the Pregnancy Planning Trial

QUESTIONNAIRE (Subscale)		Baseline Mean (SD)	34 Week Mean (SD)	Group x Time Interaction p
**BGMSRQ Total**	CGM	88.1 (13.7)	91.8 (18.4)	0.043
	Control	86.8 (14.1)	91.8 (13.9)	
**BGMSRQ Satisfaction**	CGM	33.9 (6.8)	34.5 (6.7)	NS
	Control	34.9 (6.1)	36.4 (6.3)	
**BGMSRQ Impact**	CGM	29.9 (7.7)	35.2 (7.4)	0.003
	Control	29.2 (8.1)	30.1 (7.5)	
**BGMSRQ Obstruction**	CGM	24.9 (3.6)	21.7 (5.4)	0.003
	Control	24.3 (35.1	24.5 (4.8))	
**HFS Total**	CGM	39.9 (18.9)	34.4 (18.9)	NS
	Control	42.5 (21.4)	37.2 (19.0)	
**HFS Behaviour**	CGM	17.0 (8.3)	15.7 (7.7)	0.03
	Control	16.6 (8.2)	16.0 (7.3)	
**HFS Worry**	CGM	22.9 (13.8)	18.7 (12.7)	0.039
	Control	25.9 (15.4)	25.5 (13.1)	
**PAID Total**	CGM	24.2 (14.5)	20.0 (14.3)	NS
	Control	21.5 (14.7)	19.0 (13.8)	
**SHORT FORM 12 Total**	CGM	46.0 (5.4)	46.0 (7.1)	NS
	Control	47.1 (5.4)	46.5 (5.6)	
**CGM-SAT Total**	CGM	-	166.1 (26.5)	N/A

Table S17: CGM problems and skin reactions

	Pregnant Participants		Pregnancy planning participants	
	**CGM**	**Control**	**CGM**	**Control**
	**N=103**	**N=104**	**N=52**	**N=57**
**Skin changes reported during trial**	49 (47.6%)	8 (7.7%)	23 (44.2%)	5 (8.8%)
Acute erythema	30	5	18	4
Acute edema	0	0	1	1
Chronic scabbing	4	1	3	0
Chronic dry skin	8	0	9	0
Chronic hypopigmentation	1	0	1	0
Chronic hyperpigmentation	6	0	5	0
Other, specify	28	1	7	1
If yes were any classified as severe	3	0	1	0
**Problems encountered with device**	83 (80.6%)	13 (12.5%)	44 (84.6%)	6 (10.5%)
Problem connecting transmitter to receiver	34	0	12	0
Sensor did not insert properly	25	0	19	0
Too much bleeding at the area of sensor insertion	29	0	16	0
The sensor was pulled out accidentally	20	6	9	3
The sensor was removed due to discomfort	18	2	3	1
The sensor stopped working early	27	3	20	1
Other, specify	51	5	22	1
**Reasons for not using device**	80 (77.7%)	16 (15.4%)	43 (82.7%)	4 (7.0%)
Skin irritation/pain or discomfort	21	3	7	1
Alarms too frequently	22	0	10	0
Did not provide accurate readings	19	1	11	0
Too difficult to operate	5	0	3	0
Too busy to use it	14	0	12	0
Forgot to use it	13	0	3	0
Does not provide information that is helpful for diabetes management	9	1	7	0
Device not available	8	0	12	0
Vacation	9	0	11	1
Sensor keeps coming out	7	3	5	2
Calibration issues/sensor errors	35	1	13	0
Sports/water activities	1	0	1	0
Adhesive issues	4	2	3	1
Ran out of sensors/supplies	21	0	15	0
Needed a break	35	0	19	0
Sensor insertion issues	14	1	13	0
Other, specify	37	10	10	1

Table S18: Hospital admissions in pregnant women

	CGM	Control
	**N=103**	**N=104**
Number of women with hospital admissions	28 (27.2%)	25 (24.0%)
Number of hospital admissions[§]	37	30
Number of hospital admissions per woman median (IQR)	1.0 (1.0-1.25)	1.0 (1.0)
Total number of days for hospital admission	61	42
Number of days for hospital admission median (IQR)	2.0 (1.0-2.0)	1.0 (0.0-2.0)
Reasons for admission:		
Diabetic ketoacidosis	2 (1.9%)	2 (1.9%)
Severe hypoglycaemia[‡]	2 (1.9%)	0
Hypertension	2 (1.9%)	2 (1.9%)
Obstetrical	15 (14.6%)	9 (8.7%)
Other, specify	13 (12.6%)	16 (15.4%)

[§] These data are applicable only to participants who completed at least one study visit and do not include hospital admissions <24 hours or the delivery hospital admission

[‡] Severe hypoglycaemia is from randomisation to 36 weeks gestation.

Table S19: Adverse Events (occurrences)

	Pregnant Participants		Pregnancy planning participants	
	**CGM**	**Control**	**CGM**	**Control**
	**N=109**	**N=78**	**N=24**	**N=47**
Respiratory/ENT	29 (26.6%)	22 (28.2%)	17 (70.8%)	21 (44.7%)
GI/Nausea/Vomiting	16 (14.7%)	15 (19.2%)	2 (8.3%)	14 (29.8%)
Urinary/Genital	7 (6.4%)	12 (15.4%)	0	4 (8.5%)
Cardio/Vasovagal	4 (3.7%)	4 (5.1%)	1 (4.2%)	0
Headaches/Migraines	10 (9.2%)	4 (5.1%)	1 (4.2%)	2 (4.3%)
Skin	17 (15.6%)	5 (6.4%)	3 (12.5%)	1 (2.1%)
Musculoskeletal	9 (8.3%)	6 (7.7%)	0	2 (4.3%)
Blood/Hematological	4 (3.7%)	1 (1.3%)	0	0
Neurological	6 (5.5%)	1 (1.3%)	0	0
Other	3 (2.7%)	2 (2.6%)	0	3 (6.4%)
Obstetrical	3 (2.7%)	4 (5.1%)	0	0
DKA	1 (0.9%)	0	0	0
Psychological/Psychiatric	0	2 (2.6%)	0	0

S19a: Serious Adverse Events (occurrences)

	Pregnant Participants		Pregnancy planning participants	
	**CGM**	**Control**	**CGM**	**Control**
	**N=109**	**N=78**	**N=24**	**N=47**
Respiratory/ENT	1 (0.9%)	1 (1.3%)	0	0
GI/Nausea/Vomiting	1 (0.9%)	3 (3.8%)	2 (8.3%)	1 (2.1%)
Urinary/Genital	0	1 (1.3%)	0	0
Headaches/Migraines	1 (0.9%)	0	0	0
Skin	1 (0.9%)	0	0	0
Neurological[§]	1 (0.9%)	0	0	0
Other[‡]	2 (1.8%)	0	0	0
Obstetrical	0	2 (2.6%)	0	0
DKA	1 (0.9%)	0	0	0

[§] In pregnant participant – Foot drop

[‡] In pregnant participant – Invasive ductal carcinoma of right breast and cortisol deficiency.

Figure S1: Change in HbA1c in relation to unscheduled antenatal visits

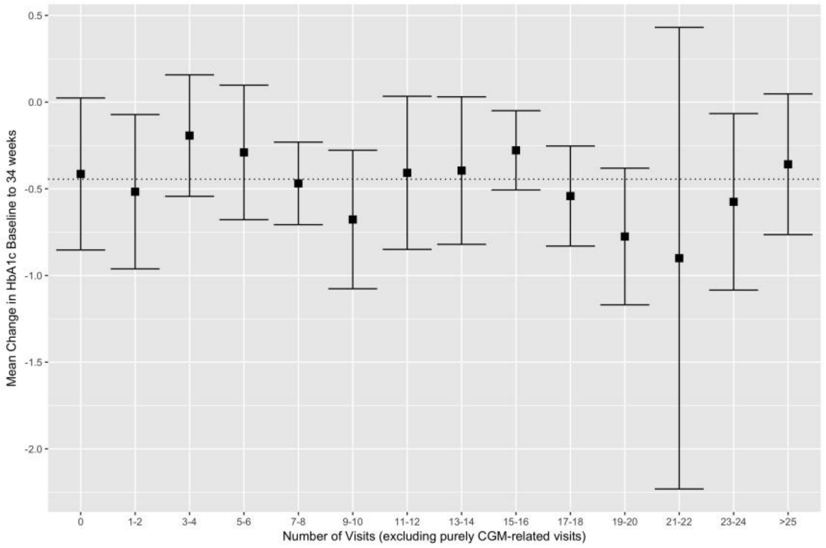

¥ This analysis of change in HbA1c by numbers of visits is an exploratory post-hoc analysis, carried out to address the possibility that the additional unscheduled visits experienced by CGM participants may have been responsible for their additional decrease in HbA1c when compared to the control participants. Unscheduled visits were classified by site investigators as only CGM-related, only diabetes-related, both CGM and diabetes-related, or other. As the number of purely CGM-related visits is almost completely confounded with treatment assignment, we excluded these CGM-only visits from the counts of unscheduled visits tallied on each participant. We formed groups based on the each participants total number of such visits (0, 1-2, 3-4, …, 23-24, >24) and within each group computed the mean change in HbA1c from baseline to 34 weeks, along with its 95% confidence interval. The results are plotted above. The number of visits had little relationship in a one-way ANOVA with these grouped visit counts (adjusted R2=6%, adjusted R2=0%; p=0.62), or with a loess fit to the actual number of visits (R2 = 2.7%, with number of equivalent parameters equal to 6). This suggests that the drop in HbA1c at the individual level was not associated with the number of visits for diabetes-alone, both diabetes and CGM, or other reasons, and therefore that additional contacts with the study in the CGM group are not likely leading to reductions in HbA1c.

Figure S2: Neonatal birthweight percentiles according to country

This shows the neonatal birthweight percentiles according to country. The number of neonates from Ireland (4) and the USA (1) were too small to be included in this post-hoc country specific analyses.

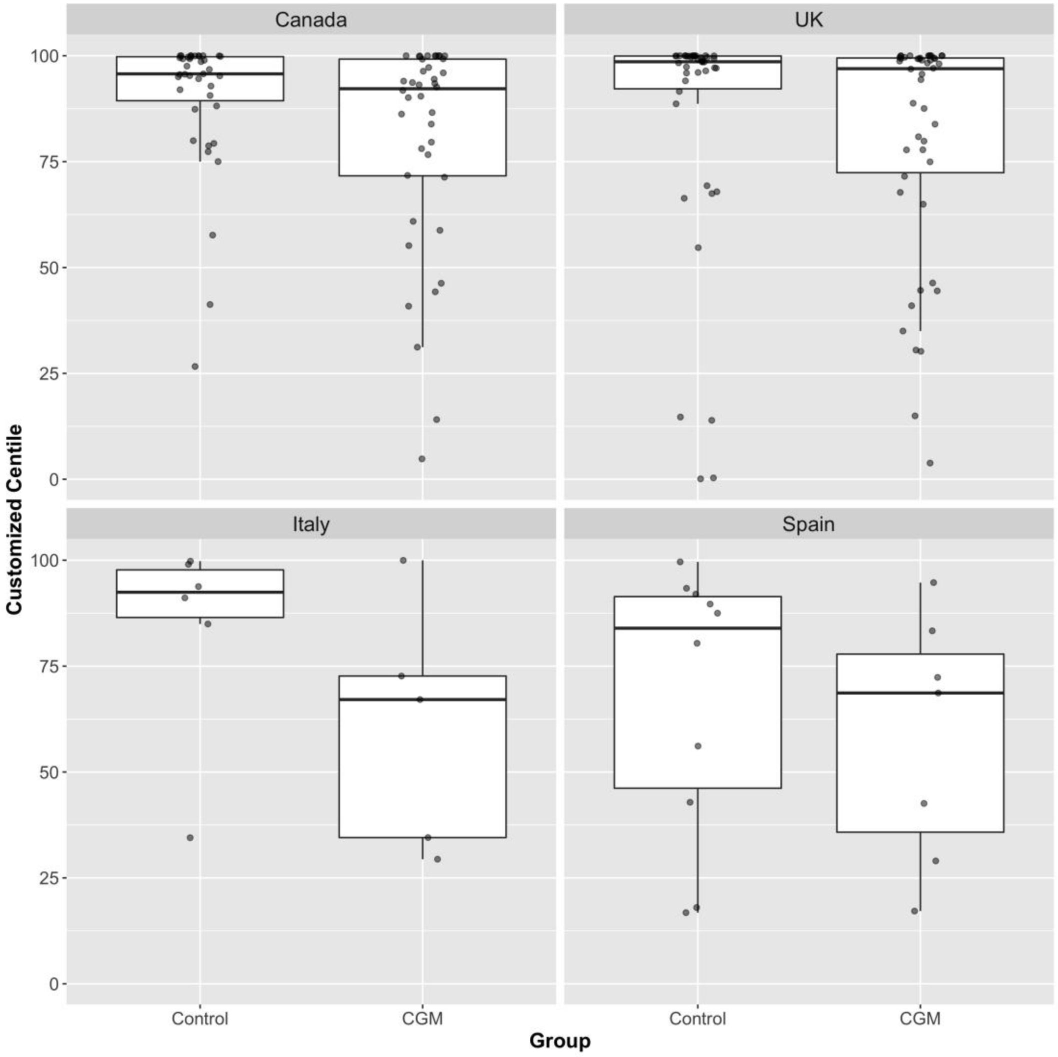

Figure S3: Neonatal Large for Gestational Age (LGA) rates according to country

The number of neonates from Ireland (4) and the USA (1) were too small to be included in this post-hoc country specific analyses. The estimated odds ratios were Canada 0.47, UK 0.49, Italy 0.28, Spain 0.54.

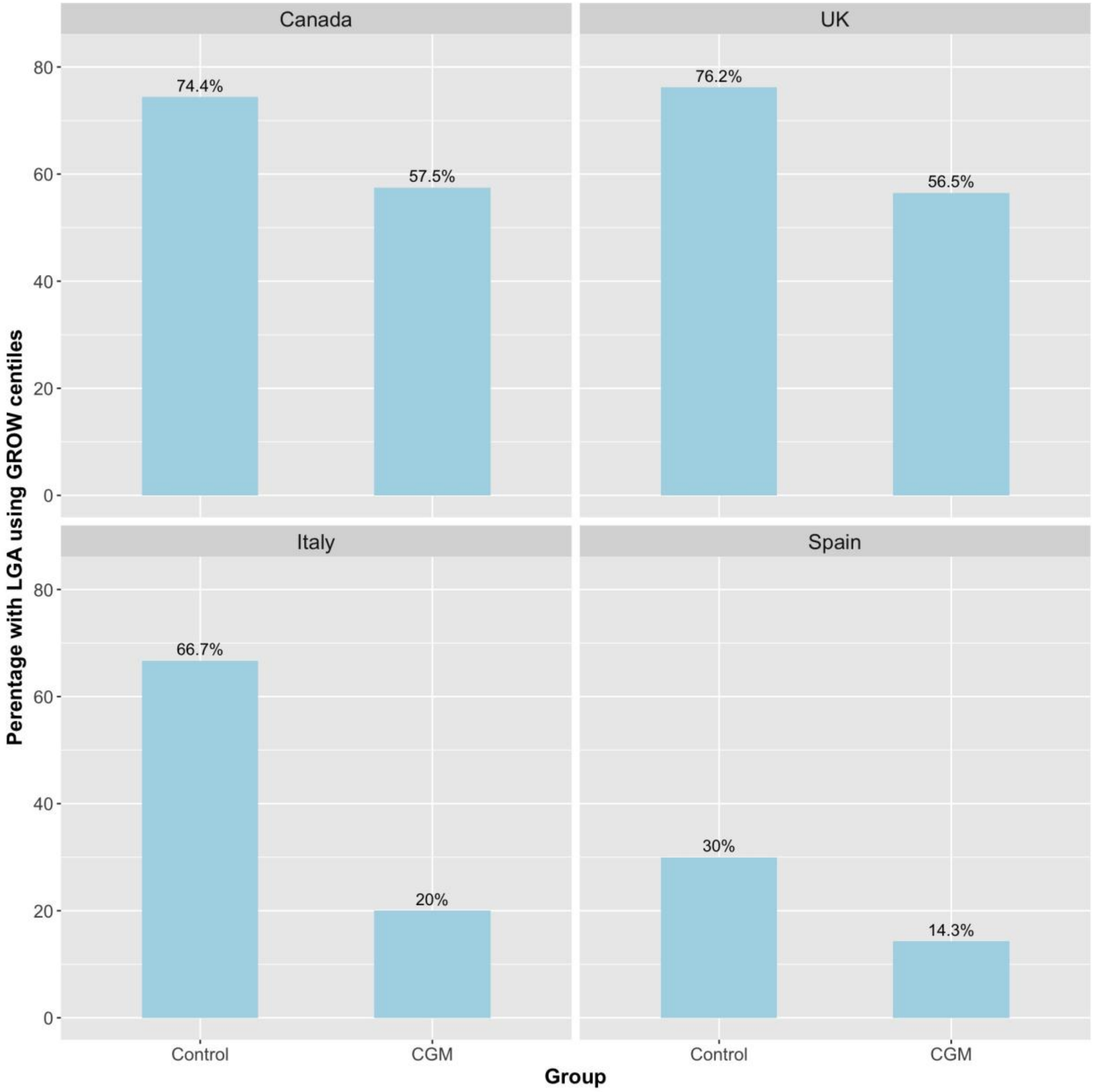

Supplement: Supplementary appendix [file mmc1.pdf]
